# Microglial P2Y12 is necessary for synaptic plasticity in mouse visual cortex

**DOI:** 10.1038/ncomms10905

**Published:** 2016-03-07

**Authors:** G. O. Sipe, R. L. Lowery,, M-È Tremblay, E. A. Kelly, C. E. Lamantia, A. K. Majewska

**Affiliations:** 1 Department of Neuroscience, University of Rochester, 601 Elmwood Avenue, box 603, Rochester, New York 14642, USA; 2 Neuroscience Graduate Program, University of Rochester, 601 Elmwood Avenue, box 603, Rochester, New York 14642, USA

## Abstract

Microglia are the resident immune cells of the brain. Increasingly, they are recognized as important mediators of normal neurophysiology, particularly during early development. Here we demonstrate that microglia are critical for ocular dominance plasticity. During the visual critical period, closure of one eye elicits changes in the structure and function of connections underlying binocular responses of neurons in the visual cortex. We find that microglia respond to monocular deprivation during the critical period, altering their morphology, motility and phagocytic behaviour as well as interactions with synapses. To explore the underlying mechanism, we focused on the P2Y12 purinergic receptor, which is selectively expressed in non-activated microglia and mediates process motility during early injury responses. We find that disrupting this receptor alters the microglial response to monocular deprivation and abrogates ocular dominance plasticity. These results suggest that microglia actively contribute to experience-dependent plasticity in the adolescent brain.

## Abstract (web summary)

Microglia play key roles during early neurodevelopment. Here the authors show that microglia are important mediators of ocular dominance plasticity (ODP). Microglia respond to monocular deprivation during the visual critical period, and disrupting microglial P2Y12 purinergic receptor abrogates ODP.

## Graphical abstract

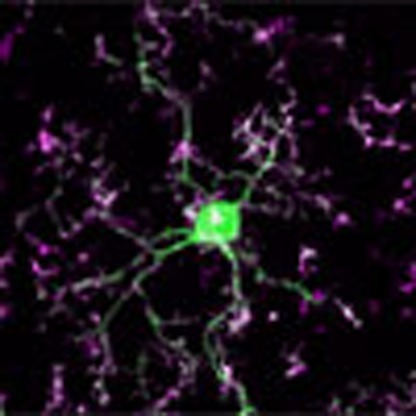

Microglia are the canonical resident immune cells of the central nervous system, with well-established roles in pathological responses^1^. Historically, it was assumed that microglia existed in a static ‘resting' state until pathological stimuli triggered their activation (migration, morphological changes, proliferation, intensified phagocytosis and release of pro- and anti-inflammatory mediators). However, landmark studies demonstrated that microglia in the healthy brain are highly motile, actively surveying the brain parenchyma^2^,^3^. This dynamic behaviour has led to a new classification of homeostatic microglial roles and has prompted new lines of research exploring potential nonpathological roles of microglia in neurodevelopment and adult circuit function. Indeed, accumulating evidence suggests that microglia perform critical roles during neurodevelopment, often using signal transduction pathways traditionally studied in neuroinflammation. For example, microglia actively shape maturing neuronal networks within the first few weeks of life in the mouse via the complement and fractalkine systems^4^,^5^. However, microglial contributions to network maintenance and plasticity at later developmental stages and the underlying mechanisms remain poorly understood. In the present study, we investigated the role of microglia in ocular dominance plasticity, a well-characterized model of adolescent experience-dependent synaptic plasticity.

Ocular dominance plasticity (ODP) was first described by Hubel and Wiesel, with the observation that monocular deprivation (MD) during a critical period of development caused a rearrangement of neuronal firing properties in the binocular visual cortex of young kittens. Cells that were initially biased to respond to inputs from the closed eye now responded more strongly to inputs from the open eye^6^. Since then, this experience-dependent plasticity has served not just to inform and improve treatment of amblyopic children, but also as a model to understand general mechanisms of activity-dependent plasticity that are applicable to other brain regions and sensory systems, both during development and throughout the lifespan. In fact, recent work has shown that animal models of several human neurodevelopmental diseases show disruptions of ODP, allowing the visual system to be used to dissect the mechanisms responsible for altered development in these disorders^7^,^8^.

Traditional studies of ODP have focused on neuro-intrinsic pathways implementing experience-dependent changes in the visual system. However, multiple lines of evidence suggest that extracellular matrix remodelling^9^,^10^,^11^,^12^,^13^ and myelination^14^ influence plasticity, implying a possible role of glial cells in this process. For example, several studies have reported that astrocytes may contribute to plastic changes during the visual critical period^15^,^16^,^17^. However, microglial involvement in ODP has remained unknown.

Microglia dynamically interact with neuronal circuits, making direct contacts with synaptic elements^18^,^19^ in a way that is modulated by experience-dependent changes in neuronal activity^19^. While the signalling pathways regulating these interactions are not well understood, there are a number of receptors expressed by microglia that can convey information regarding changes in normal brain homeostasis^1^,^20^. The microglial expression of most of these receptors has been demonstrated *in vitro*, but activation of the G_i/o_-coupled purinergic receptor, P2Y12, has been shown to powerfully alter microglial dynamics *in vivo*^21^. P2Y12 is highly sensitive to adenosine diphosphate, abundantly and selectively expressed in homeostatic microglia, rapidly downregulated following the initiation of inflammation and elicits rapid and pronounced chemotactic responses of microglial processes^21^,^22^. In addition, its expression correlates strongly with a ramified microglial morphology characteristic of healthy neurophysiology, indicating that it primarily functions during rapid responses to perturbations of brain homeostasis^21^,^23^. Given the well-documented roles of purinergic signalling in neuron–astrocyte communication^24^, we posited that P2Y12 may facilitate neuron–microglia crosstalk during ODP.

Here we demonstrate that microglia react rapidly to changes in cortical activity, increasing process arborization, reducing process motility and increasing their interactions with synaptic elements. Furthermore, disrupting P2Y12 signalling using pharmacological or genetic methods significantly inhibits microglial behaviour during MD and abrogates ODP. These findings implicate microglia as key players in the execution of plastic changes in cortical networks during experience-driven plasticity, and have implications for the understanding of microglial contributions to cognitive disorders where such functions are compromised.

## Results

**Figure 1:**
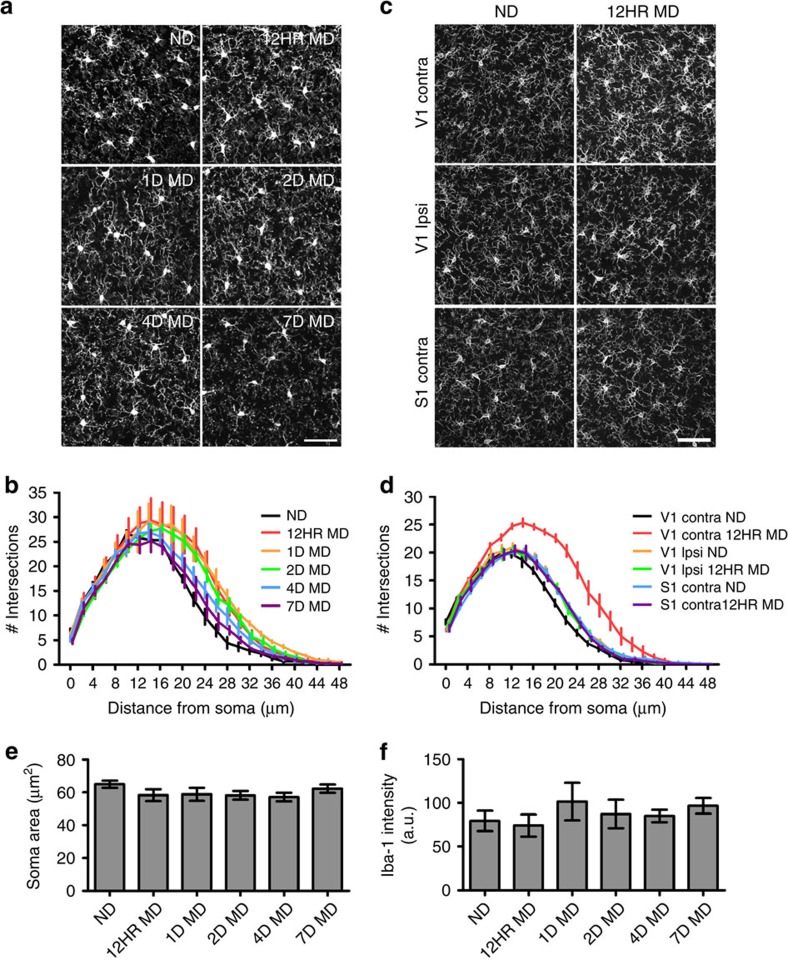
Rapid morphological changes of microglia during MD. (**a**) Images showing Iba-1 immunoreactive microglia in fixed sections of binocular visual cortex at different times following MD (MD, monocular deprivation; ND, non-deprived). (**b**) Sholl analysis of microglia at different time points following MD. Note the hyper-ramification occurring following 12 h of contralateral eye closure (*n*=3–4, two-way ANOVA, *P*<0.0001, *F*(5,40)=26.25; see Supplementary Table 1—for Holm-Sidak *post hoc* comparisons. Error bars were offset slightly between conditions for presentation purposes). (**c**) Images showing Iba-1 immunoreactive microglia in binocular visual cortex both contralateral and ipsilateral to the deprived eye, as well as primary somatosensory cortex contralateral to the deprived eye within the same animals in ND and 12 h MD conditions (note that this is a separate animal cohort from that shown in **a**,**b**,**e**,**f**). (**d**) Hyper-ramification is restricted to the contralateral binocular visual cortex (*n*=4–6, two-way ANOVA, *P*<0.0001, *F*(1,150)=379.9; see Supplementary Table 2 for Holm-Sidak *post hoc* comparisons. Error bars were offset slightly between conditions for presentation purposes). Scale bars, 50 μm. Soma size ((**e**) one-way ANOVA, *P*>0.05, *F*(5,59)=1.194) and somatic Iba-1 expression ((**f**) Kruskal–Wallis one-way ANOVA, *P*>0.05, *H*(5)=4.635) were not different at any time point after deprivation. Graphs show mean±s.e.m.

To determine whether microglia contribute to circuit changes that occur during ODP, we first examined whether these cells showed alterations in their dynamic behaviour following MD during the visual critical period (P21–P35 in mice^25^). Because microglial physiological attributes and roles are still poorly defined, we focused on well-described microglial changes associated with their pathological ‘activation'. We posited that if microglia are active participants in periods of widespread synaptic remodelling, then they may employ their traditionally pathological repertoire in the nonpathological brain. When presented with inflammatory stimuli, microglia can undergo morphological changes, increase expression of several ‘activation' markers, proliferate and translocate to the pathological locus^1^. To assay microglial morphological responses to different phases of ODP, we examined microglia in fixed sections of binocular visual cortex contralateral to the deprived eye in control animals (*n*=4) and animals that were monocularly deprived for 12 h (*n*=3), 1 day (*n*=3), 2 days (*n*=4), 4 days (*n*=4) or 7 days (*n*=3; Fig. 1a). In adolescent mice, 1–4 days of MD elicit a loss of response to the closed eye, while longer periods (4–7 days) are required to elicit an enhancement of response to the open eye. Seven days of deprivation elicits maximal plasticity^26^. While qualitative inspection of microglial morphology did not indicate obvious signs of classical activation, which is typically characterized by a retraction and thickening of processes and enlargement of the soma, we used Sholl analysis to characterize in detail more subtle changes of the microglial process arbor. This analysis uncovered a rapid, robust and significant change in microglial morphology (Fig. 1b; two-way analysis of variance (ANOVA), *P*<0.05). After as little as 12 h of MD, microglial processes became hyper-ramified, especially at a distance of 20–30 μm from the soma (Supplementary Table 1). This hyper-ramification was maintained until 4 days of MD (4D MD), when the microglial arbor returned to nearly basal dimensions. At 7 days of MD, microglial morphology was not significantly different from control. To ensure that microglial hyper-ramification was tied to ODP, we repeated the experiment in a new cohort of mice and examined microglial morphology in both the contralateral and ipsilateral binocular visual cortex, as well as the contralateral somatosensory cortex of mice that were either deprived for 12 h or left non-deprived (ND; Fig. 1c). Our analyses confirmed that hyper-ramification was limited to the contralateral binocular visual cortex (Fig. 1d and Supplementary Table 1). In addition, changes in process morphology were not accompanied by changes in soma morphology as revealed by measuring soma area (Fig. 1e), somatic intensity of Iba-1 expression (Fig. 1f) or expression of two microglial activation markers, major histocompatibility complex (MHC)-II and CD45 (Supplementary Fig. 1). This suggests that, while microglial behaviour during periods of plasticity is reminiscent of changes observed during pathology, the pathways used are distinct from traditional activation. Given the rapid microglial response, our data also point to microglial involvement in the early phase of ODP when the connections serving the deprived eye are being weakened.

**Figure 2:**
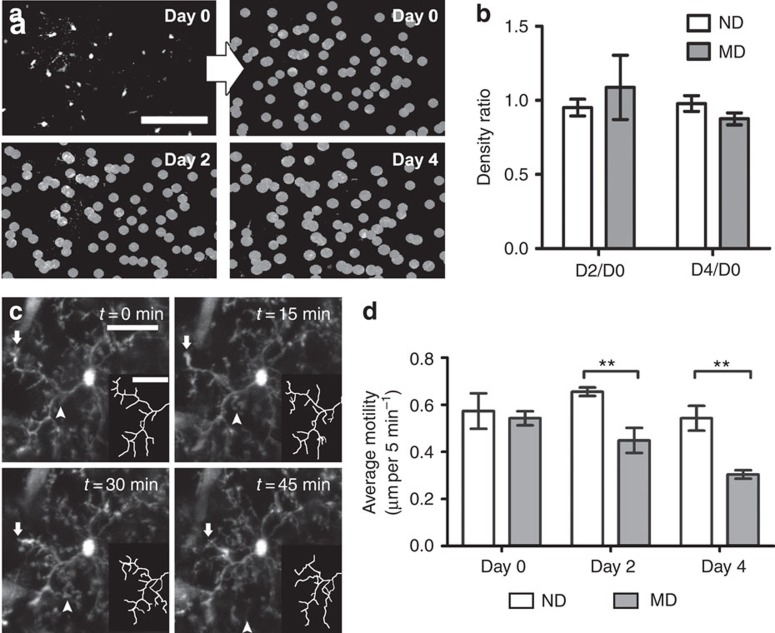
Microglial process motility changes after MD. (**a**) Example images showing microglia in binocular visual cortex chronically imaged *in vivo*. Microglial cell bodies were marked in images taken at 0, 2 and 4 days to compare microglial density over time in ND and deprived (MD) mice (mice of the MD group were deprived at the end of the Day 0 imaging session). Scale bar, 100 μm. (**b**). Quantification of microglial density as a ratio between the density observed after 2 or 4 days (D2, D4, respectively) and the density observed on day 0 (D0) in the same animal. No significant change in microglial density was observed (*n*=4–8 per group, Student's *t*-tests, *P*>0.05; D2/D0: *t*(12)=0.6946, D4/D0: *t*(8)=1.565). (**c**) Images showing time-lapse imaging of microglial motility in the binocular visual cortex. Insets show traced portions of the microglial arbor. Notice the retraction (arrow) and extension (arrowhead) of processes on the timescale of minutes. Scale bar, 25 μm. (**d**) Quantification of microglial motility (including retraction and extension of processes) on Days 0, 2 and 4 in the same animals (*n*=5 per group). Microglia were less motile in contralateral binocular visual cortex following 2 and 4 days of MD (two-way ANOVA, *P*<0.001; *F*(1,24)=17.67; Holm-Sidak *post hoc*, **P<0.01; Day(0): *t*(24)=0.472; Day(2): *t*(24)=3.16; Day (4): *t*(24)=3.65). Graphs show mean±s.e.m.

Microglia are remarkably dynamic in the intact brain. To examine their dynamic behaviour over the course of ODP, we used chronic non-invasive *in vivo* two-photon microscopy to image fluorescent microglia in the CX3CR1-GFP reporter mouse (*CX3CR1*^*GFP/+*^)^27^. Mice were prepared for chronic imaging by thinning the skull over the binocular visual cortex. Microglia were imaged at low magnification in layers 1–2 to determine density (Fig. 2a,b) and then at higher magnification every 5 min for 60 min to assay process motility (Fig. 2c,d). Animals were reimaged either after 2 or 4 days of deprivation of the eye contralateral to the imaged cortex and compared with animals that had not been deprived during the same time period. We found no change in microglial density at either 2 or 4 days of MD as compared with ND mice (Fig. 2b; also confirmed in analysis of fixed sections (Supplementary Fig. 2)). However, we found a significant change in microglial process motility after deprivation. While microglia displayed high levels of both extension and retraction on the first day of imaging in both control and experimental groups before deprivation was initiated, re-imaging after 2 or 4 days showed a reduction in the motility of microglial processes in the deprived animals only (Fig. 2d; Supplementary Movies 1 and 2; two-way ANOVA; *P*<0.01). Given the proliferation and increased process restructuring of microglia observed in pathological conditions, these results again support the notion that distinct pathways are recruited in homeostatic microglia.

**Figure 3:**
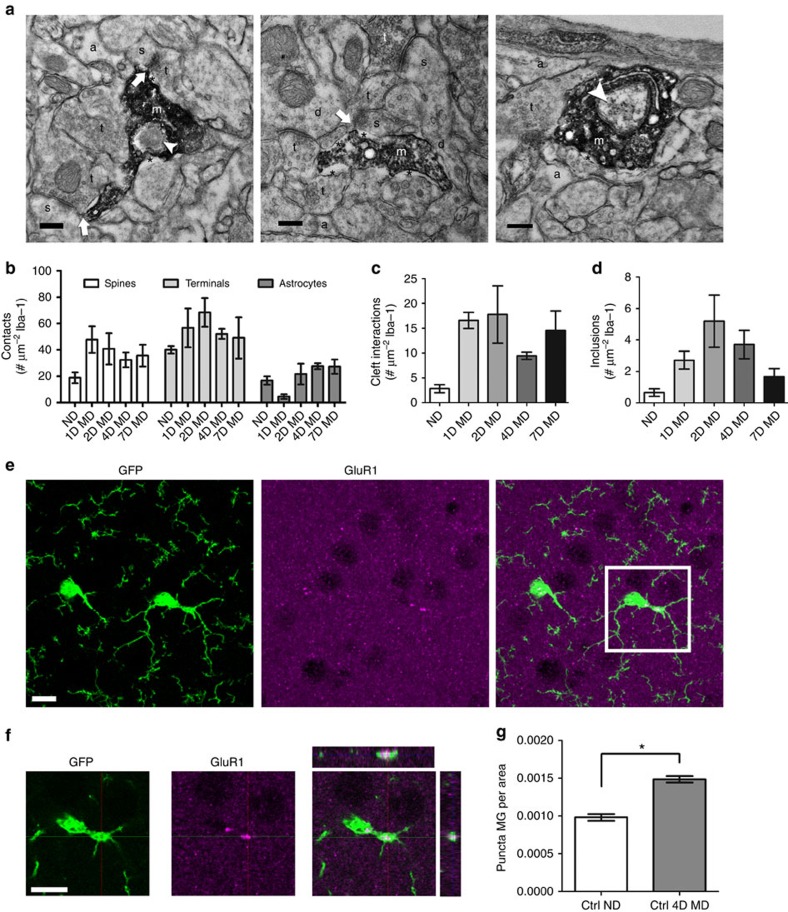
Microglial interactions with synaptic elements are altered by MD. (**a**) Representative electron micrographs of Iba-1-immunoreactive microglial processes in contralateral binocular visual cortex from 4D MD animals (a, perisynaptic astrocytic process; d, dendrite; m, microglial process; s, dendritic spine; t, axon terminal; *, extracellular space; arrow, cleft contact; arrowhead, inclusion). Scale bars, 0.2 μm. (**b**) No statistically significant difference was observed in the number of microglial contacts with dendritic spines, axon terminals or astrocytic processes (*n*=3; two-way ANOVA; *P*=0.14 for condition main effect; *F*(4,30)=1.861). (**c**,**d**) The number of microglial interactions with synaptic clefts (**c**) and the number of microglial inclusions (**d**) increase following MD (*n*=3; one-way ANOVA; *P*=0.0464; *F*(4,10)=3.578 (**c**); *P*=0.0421; *F*(4,10)=3.712 (**d**); no statistically significant effect was observed with *post hoc* analysis). (**e**) Images showing GFP (left panel), GluA1 immunoreactivity (middle panel) and a merge of the two (right panel) in fixed sections of contralateral binocular visual cortex of *CX3CR1-GFP* animals. (**f**) A single confocal *z*-plane image of the boxed area shown in **e**, showing internalization of GluA1-immunoreactive puncta by microglia (rightmost panel). Scale bars, 10 μm (**e**,**f**). (**g**) The number of internalized GluA1 puncta per unit of microglial area is increased after 4 days of MD (*n*=4,5; Student's *t*-test, *P*<0.0001, *t*(7)=7.920). Graphs show mean±s.e.m.

Microglia have been suggested to phagocytose pre- and postsynaptic elements to aid in synapse remodelling during development and plasticity^4^,^5^,^19^,^28^. To gain insight into microglial interactions with synapses after MD, we used electron microscopy (EM) to analyse microglia–synapse interactions in the binocular visual cortex at high spatial resolution (Fig. 3a). We found that Iba-1-positive microglial processes were characterized by their irregular contours and surrounding extracellular space. In addition, they frequently abutted synaptic elements such as dendritic spines, axon terminals and perisynaptic astrocytic processes. Occasionally, microglial processes were present at the synaptic cleft (Fig. 3a). To quantitatively compare these interactions between conditions, and control for the fact that large processes have the ability to interact with more brain elements and contain more inclusions, we normalized our observations to the size of the microglial process analysed. While the analysis showed a trend towards an increase in the number of microglial contacts with dendritic spines and axon terminals during the early response to deprivation (1–4 days of MD), these differences were not statistically significant (Fig. 3b; one-way ANOVA; *P*>0.05). The number of microglial contacts with synaptic clefts, however, was significantly increased by MD (Fig. 3c, one-way ANOVA, *P*<0.05) and remained elevated throughout the deprivation period (up to 7 days). Microglial processes also contained inclusions such as large vesicles, vacuoles and endosomes containing cellular elements, which may indicate active phagocytosis (Fig. 3b). Because we did not use serial sectioning EM with three-dimensional reconstruction, we cannot be sure that these inclusions represent true engulfment as opposed to partial wrapping of microglial processes around other brain elements. However, the number of processes with such inclusions was significantly elevated following deprivation (Fig. 3d, one-way ANOVA, *P*<0.05), with the highest number of inclusions observed at 2-day MD, supporting an early role for microglia in ODP during the pruning of connections from the deprived eye. To determine whether such inclusions represent true phagocytosis and contain synaptic elements, we performed immunohistochemistry for GluA1, the main subunit of the cortical glutamate receptor, in the binocular visual cortex of ND and 4D MD *CX3CR1*^*GFP/+*^ mice (Fig. 3e). GluA1 surface expression has been shown to decrease during the early phase of ODP when synapses from the deprived eye are eliminated and increase when ND eye responses strengthen^29^. Three-dimensional confocal analysis showed that, while most of the immunoreactivity for GluA1 was limited to puncta in the neuropil, some puncta were contained within microglial cell bodies and processes (Fig. 3f). A quantification demonstrated a significant increase in GluA1 puncta within the microglial cytoplasm after deprivation (Fig. 3g; Student's *t*-test; *P*<0.0001), demonstrating active engulfment of synaptic material by microglia during periods of ODP.

Next, we sought to understand the importance of the microglial contribution to ODP by disrupting microglial signalling cascades known to regulate their dynamic behaviour. We chose the purinergic receptor P2Y12 because of its high selectivity and sensitivity to ADP, which could allow microglial processes to respond to localized release of ATP under physiological conditions^30^. P2Y12 is exclusively and extensively expressed by homeostatic microglia within the brain, even distinguishing resident microglia from phenotypically similar infiltrating macrophages^23^, suggesting that P2Y12 may serve microglia-specific roles within the normal brain parenchyma.

**Figure 4:**
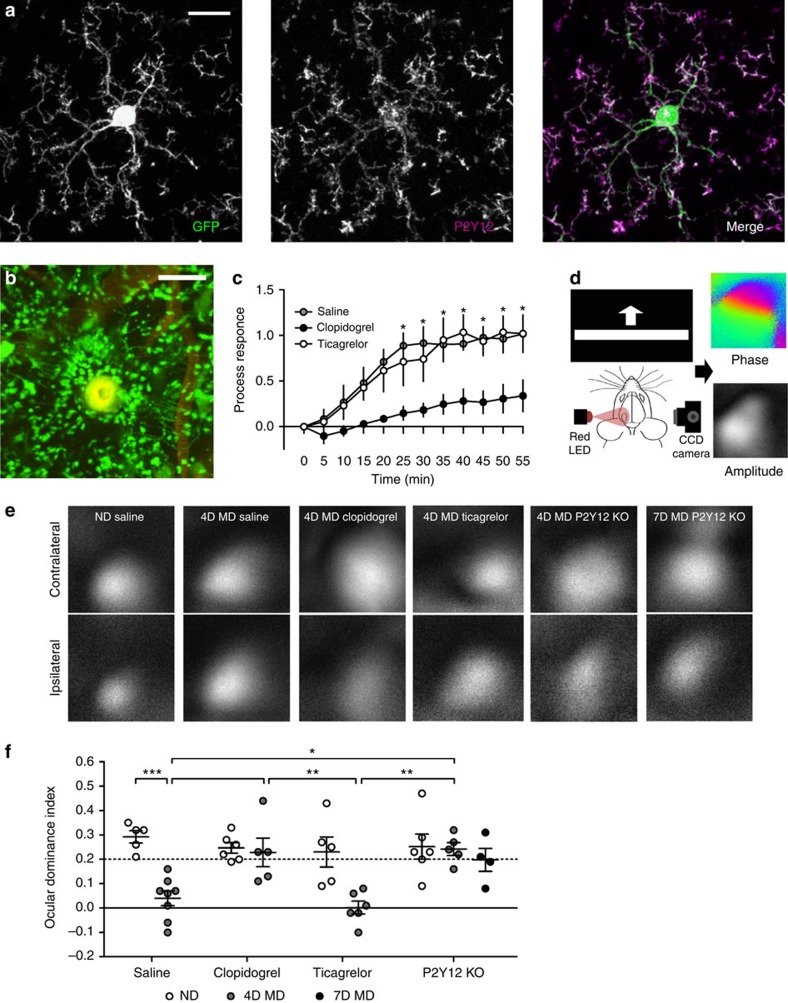
Microglial P2Y12 is necessary for ODP. (**a**) Confocal image showing immunoreactivity for P2Y12 (middle panel) in cortical microglia (left panel) from *CX3CR1*^*GFP/+*^ mice (merge in right panel; P2Y12: magenta; GFP: green). Note high P2Y12 expression on the membranes of distal microglial processes. Scale bar, 10 μm. (**b**) *In vivo* two-photon image showing the microglial response 30 min following a laser-induced injury in a saline-injected mouse. The vasculature was labelled using a retro-orbital injection of 4% tetramethylrhodamine dextran (shown in red) to ensure that the blood–brain barrier was not compromised during laser injury. Scale bar, 50 μm. (**c**) Quantification showing a statistically significant reduction in process targeting to the injury site in clopidogrel- but not ticagrelor-treated mice (*n*=3 per group, two-way ANOVA; *F*(1,48)=122.0; *P*<0.05; Holm-Sidak *post hoc*). (**d**) Schematic showing the intrinsic optical signal-imaging apparatus used in our study (left panel). Cortical responses to visual stimuli (a moving vertical bar presented on a monitor 30 cm in front of the mouse) were recorded as changes in the reflectivity of 700-nm light. This allowed the collection of phase maps (top right panel) that indicate retinotopy, and amplitude maps (bottom right panel) that indicate the strength of the cortical response. Amplitude maps obtained from stimulation of each eye independently were used to compute ocular dominance. (**e**) Representative amplitude maps obtained from contralateral and ipsilateral eye stimulations under different conditions. (**f**) Quantification of ocular dominance index shows robust shifts after MD in saline- and ticagrelor-treated mice, but no shift is observed in clopidogrel-treated and *P2Y12 KO* animals (*n*=5–7 per group; two-way ANOVA; *F*(1,28)=7.93; *P*<0.05; Holm-Sidak *post hoc*). Graphs show mean±s.e.m.

We first confirmed that microglia in mouse primary visual cortex express P2Y12 using immunostaining (Fig. 4a and Supplementary Fig. 3) (ref. 2121). P2Y12 labelling in the visual cortex was widespread and highlighted the membranes of cortical microglia expressing green fluorescent protein (GFP; *CX3CR1*^*GFP/*+^). To understand how P2Y12 expression regulates microglial behaviour both basally and during MD, we utilized the highly selective P2Y12 antagonist, clopidogrel^30^, and P2Y12 knockout mice (*P2Y12*^*KO*^) for pharmacologic or genetic P2Y12 disruption, respectively. We crossed *P2Y12*^*KO*^ animals with *CX3CR1*^*GFP*^ animals to generate *CX3CR1*^*GFP/+*^*/P2Y12*^*KO*^ animals in which we could image *P2Y12*^*KO*^ microglia *in vivo* and determine P2Y12's effects on the microglial responses. We first verified an effect of genetic P2Y12 knockout by confirming previous reports that *P2Y12*^*KO*^ animals have a reduced response to laser ablation (Supplementary Movie 3) (ref. 2121). We then sought to validate pharmacologic P2Y12 blockade in *CX3CR1*^*GFP/+*^ mice using clopidogrel, which must be catalysed by the liver to produce its active metabolite. Although this metabolite is predicted to cross the blood–brain barrier (BBB) and previous studies have shown central nervous system-specific effects after clopidogrel treatment^31^, we verified clopidogrel's cortical action in mice using the laser-ablation response (Fig. 4b). Injections of 4% rhodamine dextran into the vasculature allowed us to ensure that laser ablation did not damage nearby blood vessels, allowing clopidogrel to access the brain directly during the experiment. Clopidogrel treatment (50 mg kg^−1^, intraperitoneally (i.p.)) significantly delayed the laser ablation response relative to saline treatment (Fig. 4c; two-way ANOVA; *P*<0.01; Supplementary Movies 4 and 5), suggesting that clopidogrel's active metabolite crosses the BBB at a level that can affect P2Y12-mediated microglial responses. To confirm that these effects observed using clopidogrel are due to central blockade of P2Y12, we also used another P2Y12 inhibitor, ticagrelor, which is not predicted to cross the BBB. Indeed, in our hands, treatment with ticagrelor (10 mg kg^−1^, i.p.) did not affect the microglial response to laser ablation relative to saline treatment, showing that this inhibitor is not BBB-permeant (Fig. 4c; two-way ANOVA; Holm-Sidak *post hoc*; *P*>0.05; Supplementary Movie 6).

To determine whether disruption of microglial signalling through P2Y12 has functional consequences for ODP, we compared ocular dominance shifts following 4 days of MD in *P2Y12*^*WT*^ animals treated with saline versus *P2Y12*^*WT*^ animals treated with clopidogrel, *P2Y12*^*WT*^ animals treated with ticagrelor or *P2Y12*^*KO*^ animals. We used intrinsic optical signal (iOS) imaging to quantify responses to contralateral and ipsilateral eye input in binocular visual cortex and used the relative amplitudes of the hemodynamic response to calculate an ocular dominance index (ODI; Fig. 4d,e). As expected, ND controls treated with saline had a strong contralateral bias (Fig. 4e,f). Similarly, clopidogrel-treated, ticagrelor-treated and *P2Y12*^*KO*^-ND animals also had strong contralateral biases, suggesting that P2Y12 disruption does not disrupt early developmental network organization. Control mice treated with saline demonstrated an ocular dominance shift following 4D MD, exhibiting similar responses to the contralateral and ipsilateral visual inputs (Fig. 4e,f; two-way ANOVA; Holm-Sidak *post hoc*; *P*<0.001). However, this shift did not occur in either *P2Y12*^*WT*^ animals treated with clopidogrel or in *P2Y12*^*KO*^ animals, which retained a strong contralateral bias following 4D MD (Fig. 4e,f; two-way ANOVA; Holm-Sidak *post hoc*; *P*>0.05). Single-eye responses indicated that 4D MD results in the depression of deprived eye responses and that this effect is blocked in mice with defective P2Y12 signalling (Supplementary Fig. 4). The defect in ODP persisted in *P2Y12*^*KO*^ animals, even when the deprivation period was extended to 7 days, suggesting that disrupting P2Y12 signalling does not simply delay ocular dominance shifts. Ticagrelor-treated animals, however, showed robust shifts after 4D MD (Fig. 4e,f; two-way ANOVA; Holm-Sidak *post hoc*; *P*<0.05), suggesting that P2Y12 blockade must occur specifically in the brain to affect ODP. Given the microglia-selective expression of P2Y12 in the brain parenchyma, overall these data suggest that homeostatic microglia are necessary for ODP, and that P2Y12 signalling plays a critical role in this process.

**Figure 5:**
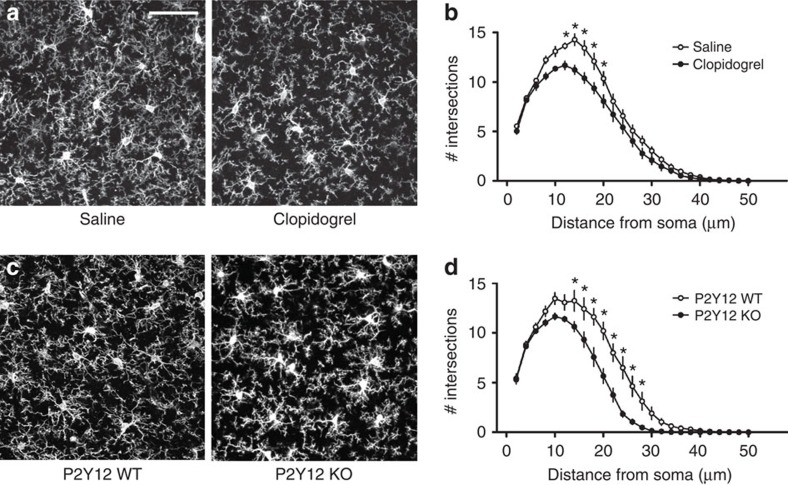
P2Y12 disruption reduces baseline microglial ramification. (**a**) Representative confocal images of microglia in the binocular visual cortex from saline and clopidogrel-treated animals. Scale bar, 50 μm. (**b**) Sholl analysis shows a reduction in ramification after clopidogrel treatment (*n*=5 per group, two-way ANOVA; *P*<0.05; *F*(1,8)=7.729). (**c**) Representative confocal images of microglia in *P2Y12 WT* and *KO* binocular visual cortex. (**d**) Microglia in *P2Y12 KOs* exhibit less ramification than in WT (*n*=4–5 per group, two-way ANOVA; *F*(1,7)=15.87; *P*<0.01; Holm-Sidak *post hoc*). Graphs show mean±s.e.m.

We next investigated the mechanisms by which P2Y12 disruption alters microglial physiology during ODP. One possible explanation for our results is that P2Y12 disruption decreases basal microglial process ramification under steady-state conditions, chronically preventing microglia from interacting with synaptic elements. In fact, previous studies have suggested a tight link between microglial ramification and P2Y12 expression^21^. Therefore, we first examined P2Y12's role in maintaining basal microglial morphology. As before, we stained microglia for Iba-1 using immunohistochemical methods to label fine processes and applied Sholl analysis on images generated with confocal microscopy to quantify microglial process ramification (Fig. 5). We found that, relative to control animals, both clopidogrel-treated and P2Y12^KO^ microglia had a modest but significant decrease in basal process ramification (Fig. 5b,d; two-way ANOVA; Holm-Sidak *post hoc*; *P*<0.05). This suggests that P2Y12 signalling contributes to, but is not necessary for maintaining microglial ramified morphology. In addition, we obtained comparable results with both pharmacological and genetic P2Y12 ablation, further confirming that systemic clopidogrel treatment disrupts P2Y12 signalling in the brain.

**Figure 6:**
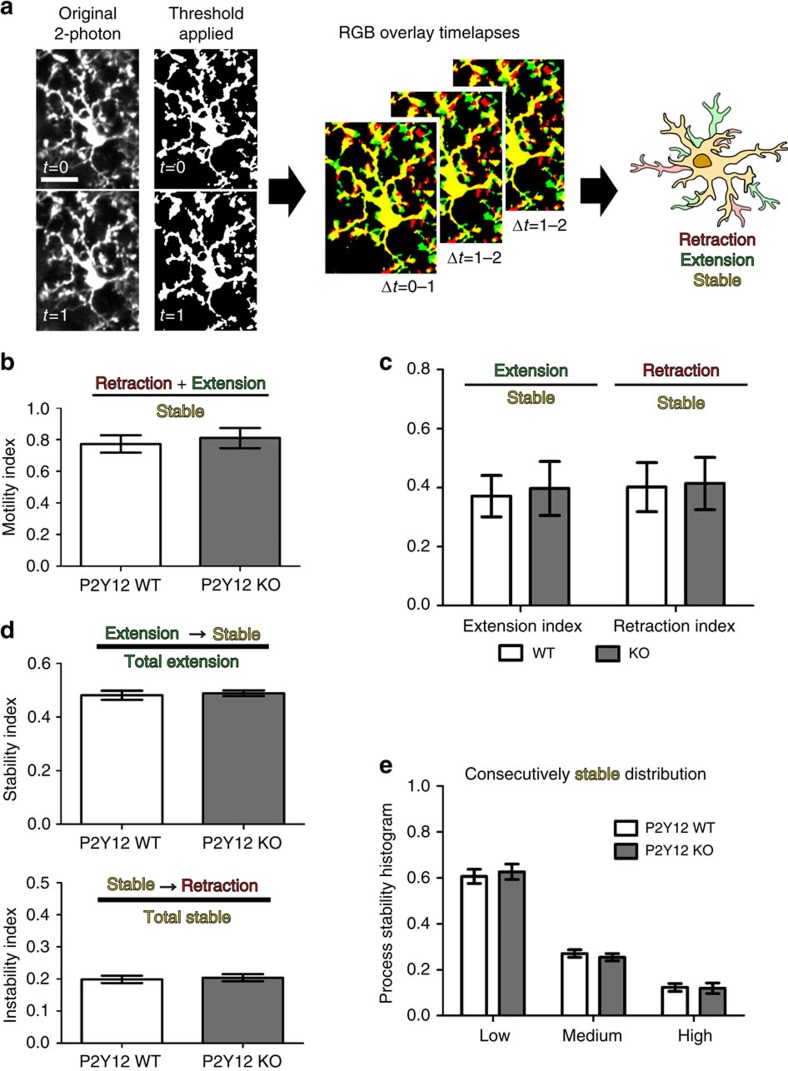
P2Y12 disruption does not affect basal microglial process motility. (**a**) Schematic showing the analysis of time-lapse *in vivo* images for microglial motility. Images are first thresholded to remove background. Consecutive images are pseudocoloured in red or green and overlayed. Yellow pixels in the overlay images are stable between the two time points, green pixels represent newly extended processes and red pixels indicate retraction. This process is repeated for all consecutive time points during the imaging session (1 h). Scale bar, 20 μm. (**b**–**e**) In binocular visual cortex, no statistically significant differences were found between P2Y12 WT and KO microglia in basal motility (**b**), extensions and retraction indices (**c**), stabilization of newly extended processes and destabilization of stable processes (**d**) or overall process stability (*n*=8 per group, Student's *t*-test; *t*(14)=0.4466 (**b**); *t*(14)=0.64 and *t*(14)=0.29 (**c**); *t*(14)=0.3786 and *t*(14)=0.3132 (**d**), two-way ANOVA, *F*=0.3044; *P*>0.05 (**e**)). Graphs show mean±s.e.m.

P2Y12 has a critical role in the rapid translocation of microglial processes to areas of injury^32^. Thus, another possible explanation for disrupted ODP after P2Y12 ablation is that P2Y12 may be necessary for basal microglial motility and consequently for synaptic surveillance. To explore this possibility, we used *CX3CR1*^*GFP/+*^*/P2Y12*^*KO*^ animals in which we could image *P2Y12*^*KO*^ microglia *in vivo* and determine P2Y12's effects on the basal motility of microglial processes. Time-lapse motility images were collected using two-photon microscopy, and process motility and stability were analysed using custom algorithms (Fig. 6a; Supplementary Movies 7 and 8). We found no differences in basal microglial process motility, retraction and extension rates, and long-term stability between *P2Y12*^*KO*^and *P2Y12*^*WT*^ animals (Fig. 6b–e; Student's *t*-test/two-way ANOVA; *P*>0.05), suggesting that P2Y12 is not critical for basal surveillance.

**Figure 7:**
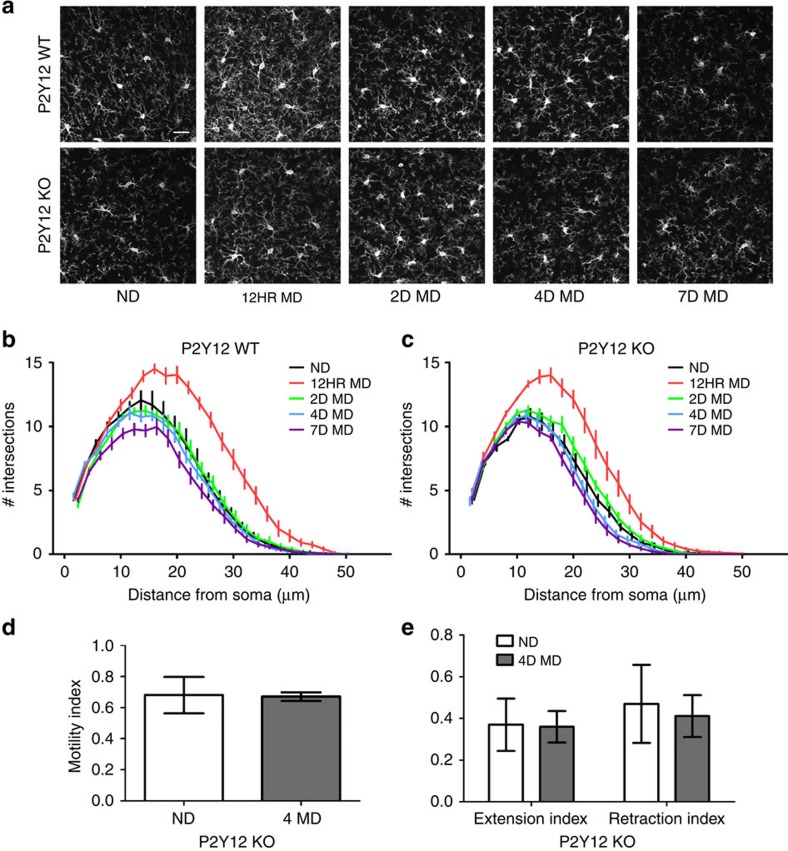
P2Y12 disruption reduces hyper-ramification during MD. (**a**) Representative confocal images showing microglia in contralateral binocular visual cortex of *P2Y12 WT* and *KO* mice before and after MD of different durations. Scale bar, 25 μm. (**b**) Sholl analysis confirms that microglia in *P2Y12* WT animals hyper-ramify following 12 h of MD (two-way ANOVA; *F*(4,25)=15.21; *P*<0.0001). (**c**) Ramification also occurred after 12 h of MD in *P2Y12* KOs (two-way ANOVA, *F*(4,25)=18.83; *P*<0.0001), although to a smaller extent than in *P2Y12 WTs*. See Supplementary Table 2 for *post hoc* statistical comparisons. Neither microglial motility (**d**) nor retraction and extension indices (**e**) are altered by 4D MD in *P2Y12 KO* mice (Student's *t*-test, *P*>0.05, *t*(7)=0.074 (**d**), *t*(7)=0.56, *t*(7)=0.14, respectively (**e**)). Graphs show mean±s.e.m.

Because we observed a chronic, modest reduction in basal microglial ramification in the *P2Y12*^*KO*^ during steady-state conditions, we wondered whether P2Y12 is necessary for the acute microglial hyper-ramification that we observed during MD. To address this possibility, we measured changes in microglial morphology during MD in *P2Y12*^*KO*^ animals versus controls (*n*=6 per group; contralateral binocular visual cortex). As before, ND *P2Y12*^*KO*^ microglia had decreased baseline ramification compared with *P2Y12*^*WT*^ controls (Fig. 7a–c and Supplementary Table 2; two-way ANOVA; *P*<0.05). Following 12 h of MD, *P2Y12*^*KO*^ microglia were still hyper-ramified, as in *P2Y12*^*WT*^ animals, although this ramification was significantly decreased compared with *P2Y12*^*WT*^ microglia (Fig. 7a–c and Supplementary Table 2; two-way ANOVA; *P*<0.05). By 2, 4 and 7 days of MD, ramifications in *P2Y12*^*WT*^ and *P2Y12*^*KO*^ microglia returned to baseline, although at 4 and 7 days MD, *P2Y12*^*KO*^ microglia had a more significant decrease in ramification than *P2Y12*^*WT*^ microglia (Fig. 7a–c and Supplementary Table 2; two-way ANOVA; *P*<0.05). Therefore, P2Y12 contributes to, but is not necessary for microglial process hyper-ramification during MD.

Given the small effect of loss of P2Y12 on MD-induced microglial hyper-ramification, we also wondered whether P2Y12 blockade may influence microglial motility after MD. We carried out *in vivo* two-photon microscopy and compared microglial motility in the contralateral binocular visual cortex of ND versus 4D MD *P2Y12*^*KO*^*/CX3CR1*^*GFP/+*^ mice. We found that, unlike in wild-type (WT) microglia, deprivation did not elicit a decrease in microglial motility in *P2Y12*^*KO*^ mice (Fig. 7d,e, Supplementary Fig. 5 and Supplementary Movie 9), suggesting that P2Y12 is necessary for some aspects of the microglial response to deprivation, such as its effects on motility.

**Figure 8:**
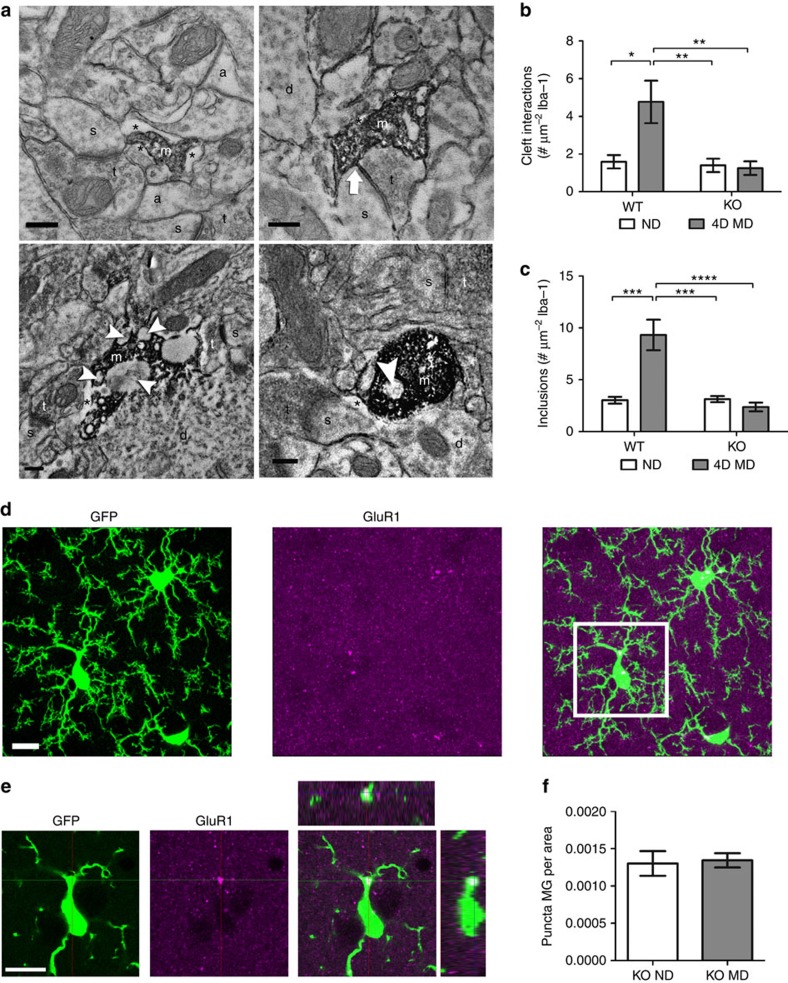
P2Y12 is necessary for microglial responses during MD. (**a**) Electron micrographs of Iba-1-immunoreactive microglial processes (a, astrocytic process; d, dendrite; m, microglial process; s, dendritic spine; t, terminal; *, extracellular space; arrow, cleft contact; arrowhead, inclusion) observed in *P2Y12 WT* (top images) and *P2Y12 KO* (bottom images) animals. Scale bars, 0.2 μm. (**b**) Quantification of microglial contacts with synaptic clefts showed significantly elevated cleft interactions following 4D MD in *P2Y12 WT* but not *P2Y12 KO* mice (*n*=5, *P*<0.05, *F*(1,16)=6.676; two-way ANOVA, Holm-Sidak *post hoc*). (**c**) The number of microglial inclusions was also increased in *P2Y12 WT* animals following deprivation but not in *P2Y12 KO*s (*n*=5, *P*<0.001, *F* (1, 16)=19.39; two-way ANOVA, Holm-Sidak *post hoc*). (**d**) Images showing GFP (left panel), GluA1 immunoreactivity (middle panel) and a merge of the two (right panel) in fixed sections of contralateral binocular visual cortex of *CX3CR1-GFP/P2Y12 KO* animals. (**e**) A single confocal z-plane image of the boxed area shown in **d** showing internalization of GluA1-immunoreactive puncta by microglia (rightmost panel). Scale bars, 10 μm (**d,e**). (**f**) The number of internalized GluA1 puncta per unit of microglial area does not change after 4 days of MD in *P2Y12 KO* mice (*n*=3,4, Student's *t*-test, *t*(5)=0.2373, *P*>0.05). Graphs show mean±s.e.m.

To test whether the effects of P2Y12 disruption on MD-induced microglial ramification affect microglia–synapse interactions, we used immuno-EM to compare *P2Y12*^*WT*^ and *P2Y12*^*KO*^ microglia during MD. Because we observed an increase in microglial contacts with synaptic clefts and phagocytic profiles following 1–4 days of MD (Fig. 3), we collected tissue from the contralateral binocular visual cortex of ND and 4D MD *P2Y12*^*WT*^ and *P2Y12*^*KO*^ animals (*n*=5, per group; Fig. 8a). Comparing *P2Y12*^*WT*^ and *P2Y12*^*KO*^ animals in the absence of deprivation revealed no difference in the basal interactions between microglia and synapses, including the prevalence of their contacts with synaptic clefts and the prevalence of intracellular inclusions (Fig. 8b,c). There were also no significant differences between *P2Y12*^*WT*^ and *P2Y12*^*KO*^ microglia with regards to contacts with dendritic spines, axon terminals and perisynaptic astrocytic processes (Supplementary Fig. 6). However, while 4 days of MD again induced an increase in microglial contacts with synaptic clefts and intracellular inclusions in *P2Y12*^*WT*^ animals (two-way ANOVA; Holm-Sidak *post hoc*; *P*<0.05 and *P*<0.001, respectively), neither of these interactions were modulated by MD in *P2Y12*^*KO*^ (*n*=5 per group; two-way ANOVA Holm-Sidak *post hoc*; Fig. 8b; *P*>0.05 and Fig. 8c; *P*>0.05, respectively). In addition, there was no increase in the density of GluA1 puncta contained within microglial cell bodies and processes in 4D MD *P2Y12*^*KO*^ animals as compared with ND *P2Y12*^*KO*^ mice (Fig. 8d–f; Student's *t*-test; *t*(5)=0.2373; *P*>0.05). These findings indicate that P2Y12 signalling could contribute to initiating microglial interactions with synaptic elements and their subsequent phagocytosis during ODP. The disruption of these behaviours in the absence of P2Y12 signalling may underlie the observed inhibition of ODP.

## Discussion

Our results suggest that microglia play a novel and critical role in the activity-dependent remodelling of neuronal networks in late development and are an integral part of the plastic machinery of the nonpathological brain. We find that microglia rapidly respond to experience-dependent changes in neuronal activity, altering their morphology resulting in a hyper-ramified state, decreasing the intrinsic motility of their processes and increasing the interactions between processes and synapses (Supplementary Fig. 7). The communication between neurons and homeostatic microglia is mediated in part by purines, signalling through the microglial P2Y12 receptor. Thus, P2Y12 is necessary not only for early phases of the injury response but is an important mediator of microglial roles in the healthy brain.

Although microglia were once considered static sentinels of the immune system, remaining inactive until a pathological stimulus prompted a response, increasing evidence suggests that microglia are dynamic participants in neurodevelopment and normal neurophysiology. Our experiments show that altering purinergic signalling in microglia can have a severe impact on ODP, an experience-dependent process occurring in the visual cortex at late developmental stages when microglia have already adopted their adult, ramified morphologies. Thus, mature microglia are crucial players in activity-dependent plasticity. Several aspects of microglial behaviour following MD should be noted (see Supplementary Fig. 7). First, microglia monitor neuronal activity, either directly or indirectly, and sense changes that occur in network activity levels. Second, microglia respond to such changes remarkably rapidly, consistent with their roles as first responders during pathological events. We observed hyper-ramification within 12 h after eye closure, a time point at which neuronal synapses have not yet undergone plastic changes and ocular dominance in cortical neurons within bV1 has not been altered. This suggests that microglia are recruited early during a plastic event and are not simply responding to plastic changes occurring at synapses. Third, the rapid response of microglia indicates that their involvement could be critical for the early phase of ODP during which deprived eye responses weaken. Last, it appears that microglia enact critical roles in synaptic plasticity without altering expression of markers canonically upregulated during pathological responses, suggesting that they possess a repertoire of signalling mechanisms critical for normal neurophysiology that are independent of pathological activation.

Our data strongly point to microglia being involved in the early phase of ODP. We observed increased phagocytosis and internalization of GluA1 receptors in microglia during this early phase when GluA1 surface expression is known to be decreased^29^, indicating that microglia may aid in the depression of deprived eye responses by pruning synapses as described during early development^4^. This is further supported by the lack of functional deprived eye depression after P2Y12 blockade. Although the presence of GluA1 receptor puncta within microglia implies that postsynaptic sites are targets for microglia after deprivation, it is likely that microglia target the whole synapse, a possibility that will have to be examined closely in the future. Interestingly, microglial interactions with synaptic clefts remain high for up to 7 days of MD, which could imply an additional microglial contribution to the strengthening of ND eye synapses. In fact, microglia were recently shown to promote dendritic spine formation through the release of brain-derived neurotrophic factor during adolescent and adult motor plasticity^33^. While we did not test this hypothesis, it is conceivable that microglia respond to MD in two phases: a rapid surveillance, interaction and phagocytosis of synaptic elements associated with decreased synaptic input, followed by a period of enhancement of synaptic outgrowth and maturation. When considering the functions of microglia in ODP, It is also important to note that, while loss of P2Y12 function occurs globally throughout the visual cortex in these models, our analysis of microglial motility, phagocytosis and synaptic contact was restricted to cortical layers 1 and 2. Synaptic changes have been described to occur rapidly in these layers^9^,^12^,^34^,^35^, and therefore microglial responses may need to be equally rapid. However, there may be additional roles for microglia in infragranular and granular layers, and these may occur at slower timescales following MD^36^.

Neuroinflammatory factors may have basal functional roles in the healthy brain, contributing to development, plasticity and normal homeostasis. These include the classical complement system^4^,^37^, signalling of the chemokine fractalkine (CX3CL1) through its microglia-specific receptor, CX3CR1 (refs 5,285,28), the traditional inflammatory cytokines interleukin 1 beta^38^ and tumour necrosis factor alpha^39^,^40^, as well as recognition molecules such as MHC-I (ref. 41^41^). Here we show that purinergic signalling through P2Y12 is an important pathway in nonpathological microglia that is critical for microglia–neuron interactions during plasticity.

As a consequence of being the primary substrate for cellular metabolism, intercellular ATP signalling is evolutionarily old with highly conserved functions in monitoring tissue metabolic homeostasis^24^. The high intracellular concentration and extensive extracellular regulation of ATP produces a strong concentration gradient where sudden increases in extracellular ATP convey pathological information such as compromised membrane integrity of nearby cells^2^. Thus, most work investigating purinergic signalling in microglia focuses on purinoceptors that are stimulated by high levels of extracellular ATP and respond to these concentrated changes in extracellular purines. However, ATP signalling also has roles in developmental network synchrony^42^, astrocytic communication^43^ and synaptic inhibition^44^, suggesting that ATP may also act as a non-inflammatory signal to microglia. P2Y12 expression is a hallmark of nonpathological microglia, and many studies show that significant levels of P2Y12 expression are limited to microglia within the brain (Fig. 4 and Supplementary Fig. 3) (refs 23,45,46,4723,45–47). P2Y12 expression can distinguish resident microglia from infiltrating monocytes, suggesting that its signalling is important for microglial functions in the physiological brain^23^. Although P2Y12 mediates early microglial process extension towards sites of injury, we demonstrate here that P2Y12 also contributes to microglial roles in synaptic plasticity. Outside of the brain, P2Y12 is highly expressed by platelets in the blood and a subset of macrophages^23^,^48^. Our experiments using a P2Y12 inhibitor that does not cross the BBB—ticagrelor—demonstrate that blocking P2Y12 peripherally does not affect plasticity. Taken together, our studies suggest that P2Y12 signalling may represent a common mechanism that enables microglia to respond to altered synaptic activity either during injury or nonpathological changes in network function.

While our study shows that purinergic signalling through the P2Y12 receptor is a crucial mediator of synaptic refinement, it does not address the processes that lead to ATP release, downstream signalling in microglia or the mechanism mediating microglial actions at synapses. The rapid morphological hyper-ramification and phagocytosis, along with a literature that implicates microglia in the phagocytosis of synaptic elements during development^4^,^28^, suggests that P2Y12 may be part of a pathway that targets synapses for monitoring and phagocytosis during ODP (Supplementary Fig. 7). Recent work has suggested that neuronal N-methyl-D-aspartate (NMDA) receptor stimulation can provoke local neuronal ATP release that is sufficient to induce transient microglial process extension via P2Y12 (refs 49,5049,50). Whereas during injury, long-range signalling of pathological events requires NMDA-mediated intercellular calcium waves to activate microglia distant to the lesion, it is possible that during ODP^51^, widespread NMDA signalling at synapses is sufficient to release ATP locally and trigger the recruitment of nearby microglial processes. Interestingly, the first phase of ODP requires NMDA receptor-dependent long-term depression (LTD)^52^. However, because hyper-ramification also occurs in P2Y12^KO^ microglia, it is unlikely that neuronal ATP release is the global signal that allows microglia to sense changes in neuronal activity during ODP. This suggests that the initial hyper-ramification does not target microglial processes to synapses but instead serves as an initial alert, allowing microglia to adopt a state that aids in their subsequent actions at synapses. Once microglia have been alerted through another mechanism, P2Y12 signalling may then be involved in the extension of processes specifically towards synapses undergoing LTD.

Thus, P2Y12 signalling would result in the targeting of microglial processes to synaptic elements requiring elimination, at which point other pathways including those mediating phagocytosis could become active. This may include pathways that involve cross-membrane communication between deprived synapses and microglial processes, such as MHC-I/PirB signalling, which has known roles in ODP and is thought to require microglial involvement^53^. Microglia can also potentiate NMDA receptor activity through glycine release^54^ and promote LTD via NADPH oxidase^55^, suggesting that microglia may have additional roles in ODP besides phagocytosis once they target a synapse. These data advocate the possibility that microglia sense and facilitate initial periods of NMDA-mediated LTD in the weakened contralateral inputs via P2Y12-dependent synapse targeting. However, it is also possible that microglia respond to changes in neuronal firing patterns indirectly via signalling through other glial elements, such as astrocytes. This possibility is supported by evidence suggesting that astrocyte–microglia communication dictates which synaptic elements should be pruned during thalamo–cortical network refinement,^56^ and that microglia respond to changes in ionotropic neurotransmission via the intermediary release of ATP by astrocytes^20^. Future studies will be needed to test whether weakened, deprived eye synapses release ATP during LTD, and how their ATP release contributes to the potentiation of ND eye connections.

Microglia in the developing brain have significantly different phenotypes from microglia in the mature brain, and could have different functions. Thus, the question remains as to whether and how mature, ramified microglia participate in neurophysiological brain function. While some studies suggest that without mature microglia or microglial release of trophic factors learning-dependent spine formation and subsequent behavioural improvement are significantly decreased^33^, others show that cognition and motor performance are not altered by pharmacological elimination of mature microglia^57^. Here we show that altering signalling through a single receptor in ramified microglia, either genetically or pharmacologically, can severely inhibit neuronal plasticity, suggesting that ramified microglia do, in fact, play critical roles during activity-dependent plasticity. While more work will be needed to elucidate the precise contributions of microglia to different physiological processes within different brain areas and at different points in the lifespan, our study highlights the dynamic abilities of these cells to influence neuronal function.

## Methods

### Animals

Experimental protocols were carried out in strict accordance with the University of Rochester Committee on Animal Resources and conformed to the National Institutes of Health Guidelines. Experiments were conducted on mice with a C57/Bl6 background during the visual critical period (P25–35). All experiments and analyses were carried out blind to genotype and manipulation. Sex distribution is available in Supplementary Table 3.

### Monocular deprivation

Animals were separated into ND or MD cohorts on P28±1. MD animals were anaesthetized (isoflurane, 5% induction, 3% maintenance) and right eyelids resected and sutured together. Eyes were not reopened except for intrinsic signal-imaging experiments. All analyses were carried out contralateral to the deprived eye (that is, left hemisphere), unless stated otherwise.

### Histology

Whole brains were collected following transcardial perfusion and overnight post-fixation with paraformaldehyde (4%). Tissue was cryoprotected and coronal sections were cut on a freezing microtome (Microm; Global Medical Instrumentation, Ramsey, MN) at 50-μm thickness. Sections were processed free-floating at room temperature (RT). Briefly, sections were rinsed and endogenous peroxidase activity and nonspecific binding blocked. Sections were then incubated in a primary antibody solution (24 h, 4 °C, anti-Iba-1, 1:1,000, Wako #019-19741; anti-MHC-II, 1:7,500 BD Pharmingen #556999; anti-CD45, 1:1,000, Serotec #MCA1031G; anit-GluA1, 1:500, EMD Millipore #PC246; anti-P2Y12, 1:1,000, courtesy David Julius) followed by secondary antibody solution (4 h, RT, Alexa-Fluor 488, 594 or 647, 1:500, Invitrogen), mounted and coverslipped.

For examination of microglial ramification and density, areas contained entirely within binocular primary visual or primary somatosensory cortex were identified and imaged on a Zeiss LSM 510 confocal microscope (Carl Zeiss, Thornwood, NY). For each section, a z-stack encompassing the entire thickness of the section was collected with a z-step of 1 μm at magnifications of × 20 and × 40. Analysis was performed offline in ImageJ. Z-stacks were smoothed and compressed into a single z-projection. For analysis of ramification, microglia in × 40 images whose entire process arbor was contained within the image were individually selected and cropped into a new image. Each image was thresholded to generate a binarized outline of the process arbor, filtered to remove artefacts and analysed with an automated Sholl analysis plugin (kindly provided by the Anirvan Ghosh laboratory, UCSD). For analysis of microglial activation, sections stained with either MHC-II, CD45 or Iba-1 were imaged on BX51 Olympus scope (Olympus, Tokyo, Japan) mounted with a Spot Pursuit RT colour digital camera (Diagnostic Instruments, Sterling Heights, MI) at the same exposure settings. For quantitative analysis of microglial soma size and Iba-1 expression, microglial somas were outlined manually in ImageJ. The area- and background-subtracted mean intensity was measured and averaged for all microglia in a single animal. Microglial density was quantified on × 20 confocal images by manually counting the number of Iba-1-positive microglial cell bodies present within a measured area.

For quantification of colocalization of alpha-amino-3-hydroxy-5-methyl-4-isoxazolepropionic acid (AMPA) receptor subunits within microglia, a two-channel z-stack encompassing a 10-μm section of tissue was collected with a z-step of 0.5 μm at a magnification of *×* 100 using confocal microscopy. Analysis was carried out offline in ImageJ. For each channel, z-stacks were smoothed and thresholded. For accurate, objective thresholding, the triangle method of auto-thesholding was selected^58^. The number of GluA1 puncta localized within microglial cell bodies and processes was determined by multiplying thresholded, binarized z-stacks in each channel and quantifying the number of particles larger than 1 pixel. Colocalization was determined as the number of internalized puncta divided by the total area occupied by microglia.

### Two-photon microscopy

A custom two-photon laser-scanning microscope was used for *in vivo* imaging (Ti:Sapphire, Mai-Tai, Spectraphysics; modified Fluoview confocal scan head, × 20 lens, 0.95 umerical aperture, Olympus). Excitation was achieved using 100-fs laser pulses (80 MHz) at 920 nm with a power of ∼40 mW measured at the sample after the objective lens. For baseline motility experiments, a 580/180 (GFP) filter was used. For laser ablation experiments, a 565 dichroic and 580/180 (GFP), 578/105 (rhodamine) filters were used. For microglial density and baseline motility, mice were anaesthetized with Avertin (200 mg kg^−1^, i.p.). For P2Y12 disruption experiments, mice were anaesthetized with a mixture of fentanyl (0.05 mg kg^−1^, i.p.), midazolam (5.0 mg kg^−1^, i.p.) and dexmetatomadin (0.5 mg kg^−1^, i.p.). During surgery and imaging, body temperature was maintained at 37 ^o^C. Imaging was carried out using × 1–10 digital zoom and 0.5–1-μm z-step. Time-lapse imaging used 4–5-min imaging intervals over 1–2 h. Image analysis was carried out offline in ImageJ or in Matlab using custom algorithms.

Microglial density was analysed by individually marking all cell bodies present in the whole-field z-stack. The image channels were then separated to generate a z-stack containing solely the cell-body markers. The z-stack was compressed into a z-projection and the number of cell-body markers counted. This total cell number was then divided by the combined area of all sections of the z-stack to generate a measure of cell density.

Microglial motility after MD was measured by individually tracing and measuring the length of the same individual microglial processes at 5-min intervals over a 1-h period. For each animal, analysis was performed on a single microglial process, defined as a process whose primary and secondary processes were clearly visible in their entirety in all 12 z-stacks over the hour period. The sections encompassing this process were identified for each of the 12 z-stacks and compressed into a single image z-projection. Beginning with the z-stack representing the 0-min time point, each individual process was traced, measured and numbered. This process was repeated for each z-stack in the hour period. Process motility was then calculated using the following formula: |(length of processX @timeB−length of processX @timeA)/5 min|. Because these animals had been anaesthetized using avertin, time-lapse imaging was not stable enough to allow for a more automated analysis of motility (see below).

Microglial motility for *P2Y12 KO* animal comparison was performed in ImageJ and Matlab using custom algorithms. Time-lapse imaging consisting of 40-μm-deep z-stacks were collected every 4 min, 12 times, for a total of 48 min. Single-image 15-μm Z-projections were created for each time point, and lateral motion artefact was corrected. Z-projections were then thresholded and time points compared. RGB overlays were created for each pair of time points (0–4 min, 4–8 min and so on) such that red pixels (retraction) were present in the first time point, but absent in the second. Green pixels (extension) were present in the second, but not in the first, and yellow pixels (stable) were present in both time points. Motility index (representing change over 4 min) was then calculated for each RGB overlay as the sum of all extension and retraction pixels divided by yellow pixels and averaged across all RGB overlays. A custom MatLab programme was used to compare pixels across multiple RGB overlays (for example, 0–4 min versus 4–8 min). Stability index was calculated as the proportion of extension pixels (green) in one RGB overlay that became stable (yellow) in the subsequent overlay divided by the total extension (green) pixels in the first overlay. Conversely, an instability index was calculated as the proportion of stable (yellow) pixels in one overlay that became retracted (red) in the subsequent overlay divided by the total stable (yellow) pixels in the first overlay. Finally, the stability histogram compared the relative stability over multiple overlays of pixels that became stable at any time point in the imaging session (but omitting all pixels that were stable for the entire imaging session, generally somas or primary processes). For both sets of motility experiments, we did not observe trends towards increased or decreased motility when comparing the first three imaging time points to the last three imaging time points.

Laser ablations were produced by performing a point scan for 3–4 s at 780 nm using ∼75 mW at the sample. Quantification of microglial response to laser ablation was measured as previously described^2^ by calculating the number of microglial processes entering from an outer radius (*Y*, ∼80 μm from ablation) into an inner radius (*X*, ∼40 μm from ablation) as a function of time. Time-lapse z-projections were produced and thresholded to normalize background variability. The total number of pixels in *X* and *Y* were measured across time (*Rx*(*t*); *Ry*(*t*)) and ablation response was calculated using the equation *R*(*t*)=(*Rx*(*t*)−*Rx*(0))/*Ry*(0). The vasculature was labelled using a retro-orbital injection of 4% tetramethylrhodamine dextran in saline to ensure that the BBBwas not compromised by laser injury.

### Electron microscopy

EM was performed as previously described^59^,^60^. Briefly, the primary visual cortex was prepared with 0.1% sodium borohydride (in 0.1 M PBS), washed and processed freely floating following a pre-embedding immunoperoxidase protocol. Sections were incubated with anti-Iba-1 (48 h, RT, 1:1,000, Wako), goat anti-rabbit IgG conjugated to biotin (2 h, RT, Jackson Immunoresearch) and streptavidin-horseradish peroxidase (1 h, RT, Jackson Immunoresearch). Immunoreactivity was visualized with diaminobenzidine (0.5 mg ml^−1^) and H_2_O_2_ (0.03%; DAB Peroxidase Substrate Kit; Vector Laboratories).

Sections for EM were post-fixed (1% OsO_4_), dehydrated, treated with propylene oxide, impregnated in Durcupan (24 h, RT, EMS), mounted between ACLAR-embedding films (EMS) and cured (48 h, 55 °C). Binocular primary visual cortex (bV1) was excised and ultrathin sections (60–80 nm) were cut (Reichert Ultracut E) and images generated (Hitachi 7650 Transmission Electron Microscope, Gatan Erlangshen camera). Approximately 50–80 pictures were randomly taken at × 30 000 in Layer 2 of bV1 (∼10 μm from pial surface) corresponding to ∼1,000 μm^2^ of neuropil per animal. Cellular profiles were identified using a series of criteria previously defined in single-ultrathin sections^19^,^61^. All subcellular profiles that were difficult to identify were classified as ‘unknown'. For quantification, measurements were normalized to the area of individual microglial processes to account for the fact that large processes have the ability to interact with more brain elements and contain more inclusions than smaller processes.

Elements were classified as follows. Dendritic shafts cut longitudinally were recognized by their irregular contours, elongated mitochondria parallel to their central axis, frequent protuberances (spines, filopodia and small branches) and synaptic contacts with axon terminals (see below). When cut transversally, dendritic shafts were identified by their rounded morphology, frequent occurrence of mitochondria and microtubules, and distinguished from unmyelinated axons by their larger diameter. Dendritic spines cut longitudinally often protruded from dendritic shafts, displayed rounded morphologies and were generally free of mitochondria. They were characterized primarily by the presence of electron-dense accumulations (postsynaptic densities) at sites of synaptic contact with axon terminals.

Axonal terminals were distinguished from other subcellular profiles based primarily on the presence of 40-nm-diameter synaptic vesicles, with rounded to elongated morphologies, but also of synaptic contacts with dendritic shafts and spines. Axon terminals generally contained mitochondria.

Protoplasmic astrocytes were recognized as electron-lucent structures seen to encase and wrap around other neuropil structures. As a result, astrocytes maintained irregular and angular shapes, distinguishing them from other neuronal profiles having a characteristic rounded shape. Perisynaptic astrocytic processes were defined by their direct contacts with axon terminals and/or dendritic spines.

Microglial processes displayed irregular contours with obtuse angles, distinctive long stretches of endoplasmic reticulum visible through the 3,3′-diaminobenzidine staining, electron-dense cytoplasm, numerous large vesicles, occasional multivesicular bodies, vacuoles or cellular inclusions and distinctive surrounding extracellular space^19^, distinguishing them from astrocytes, oligodendrocytes or NG2-positive glial cells.

Quantitative analyses of synaptic cleft interactions, element contacts and inclusions were calculated by dividing the number of events per Iba-1-labelled process by the area of the process (μm^2^) and averaging across processes (50–88 per animal). For measurement of microglial process areas, individual microglial processes were traced with the freehand line tool in ImageJ. A Grubb's outlier test was used once to remove outliers (these were exceptionally small process whose normalization yielded a very large number).

### iOS imaging

*P2Y12*^*WT*^ animals were injected with clopidogrel (50 mg kg^−1^, i.p., Mylan S.A.S), ticagrelor (dose, i.p. kindly supplied by Lundbeck Research USA Inc.) or saline daily for 4 days beginning the day of deprivation. Following 4 days of ND or MD, animals were re-anaesthetized with isoflurane and chloroproxithene (2 mg kg^−1^), and sutured eyes reopened. The skull over contralateral visual cortex was exposed, cleared, covered with agarose (0.5%) and sealed with a coverslip. Animal anaesthesia was maintained with isoflurane (0.75%) throughout imaging.

A custom-made iOS imaging set-up was used to record activity in the visual cortex during presentation of a visual stimulus (DALSA 2M30 CCD)^62^. The cortex was illuminated with 550-nm light to identify vasculature and 700-nm light for iOS collection. Images of the left visual cortex were collected continuously, while either the ipsilateral or contralateral eye was stimulated by white horizontal square-wave bar gratings on a black background moving upwards (90^o^) or downwards (270^o^) at a frequency of 8^o^ s^−1^ for 6 min (30 cm from eyes). Visually evoked responses were collected for each eye individually. The normalized amplitude of the fast Fourier transform component of the intrinsic signal was averaged for each eye from responses to both stimulus directions and compared between eyes offline using MatLab to determine ocular dominance. An ODI was computed using the following equation: ODI=(average contralateral response−average ipsilateral response)/(average contralateral response+average ipsilateral response).

### Code availability

Matlab code for motility analysis is available on request from the corresponding author.

### Statistics

Statistical comparisons were made between animal cohorts using the Prism VI statistical analysis software (GraphPad, La Jolla, CA). All *n*-values represent individual animals, and are comparable to standard *n* values used in similar experiments in the literature. All values reported are the mean± s.e.m. For all analyses, *α*=0.05. Two-tailed unpaired Student's *t*-tests and one-way or two-way ANOVAs with Holm-Sidak *post hoc* comparisons were used to compare cohorts where appropriate.

## Additional information

**How to cite this article:** Sipe, G. O. *et al.* Microglial P2Y12 is necessary for synaptic plasticity in mouse visual cortex. *Nat. Commun.* 7:10905 doi: 10.1038/ncomms10905 (2016).

## Supplementary Material

Supplementary Information Supplementary Figures 1-7 and Supplementary Tables 1-3

Supplementary Movie 1 Microglial motility imaged *in vivo* in the binocular visual cortex of a non-deprived mouse every 5 minutes for 1 hour.

Supplementary Movie 2 Microglial motility imaged *in vivo* in the binocular visual cortex of a 2 day deprived mouse every 5 minutes for 1 hour.

Supplementary Movie 3 Microglial response to a laser ablation imaged in a P2Y12 KO mouse. Images were taken every 5 minutes for 1 hour.

Supplementary Movie 4 Microglial response to a laser ablation imaged in a saline-injected control mouse. Images were taken every 5 minutes for 1 hour.

Supplementary Movie 5 Microglial response to a laser ablation imaged in a clopidogrel-injected mouse. Images were taken every 5 minutes for 1 hour.

Supplementary Movie 6 Microglial response to a laser ablation imaged in a ticagrelor-injected mouse. Images were taken every 5 minutes for 1 hour.

Supplementary Movie 7 Microglial motility imaged *in vivo* in a P2Y12 WT control mouse every 4 minutes for 48 minutes.

Supplementary Movie 8 Microglial motility imaged *in vivo* in a P2Y12 KO mouse every 4 minutes for 48 minutes.

Supplementary Movie 9 Microglial motility imaged *in vivo* in a P2Y12 KO mouse every 5 minutes for 1 hour after 4 days of MD.
