# Supplementary material for: Microglial P2Y12 is necessary for synaptic plasticity in mouse visual cortex
Source: Nat Commun. 2016 Mar 7;7:10905. doi: 10.1038/ncomms10905 (PMC4786684; doi:10.1038/ncomms10905)
Supplement: Supplementary Information — Supplementary Figures 1-7 and Supplementary Tables 1-3 [file ncomms10905-s1.pdf]

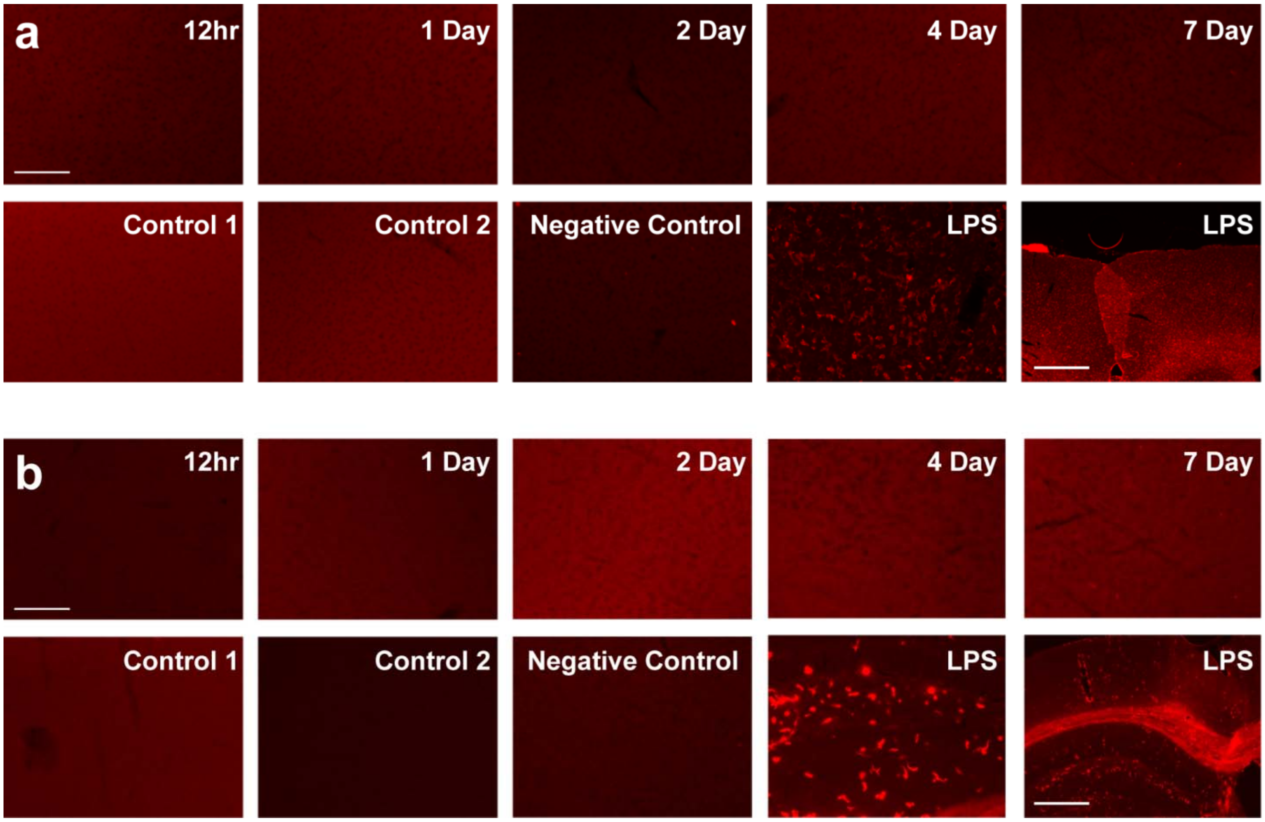

Supplementary Figure 1. Microglia do not show signs of classical immune activation following MD

a-b. Images showing immunoreactivity for MHCII (a) and CD45 (b) in fixed sections of binocular visual cortex at different times following MD (control 1 and 2 = ND). A negative control was included where the primary antibody was omitted. No expression of either marker was noted in ND or MD conditions. Lipopolysaccharide (LPS) i.p. injection robustly upregulated the expression of both markers. Scale bar = 100 μm (first panel) or 500 μm (last panel).

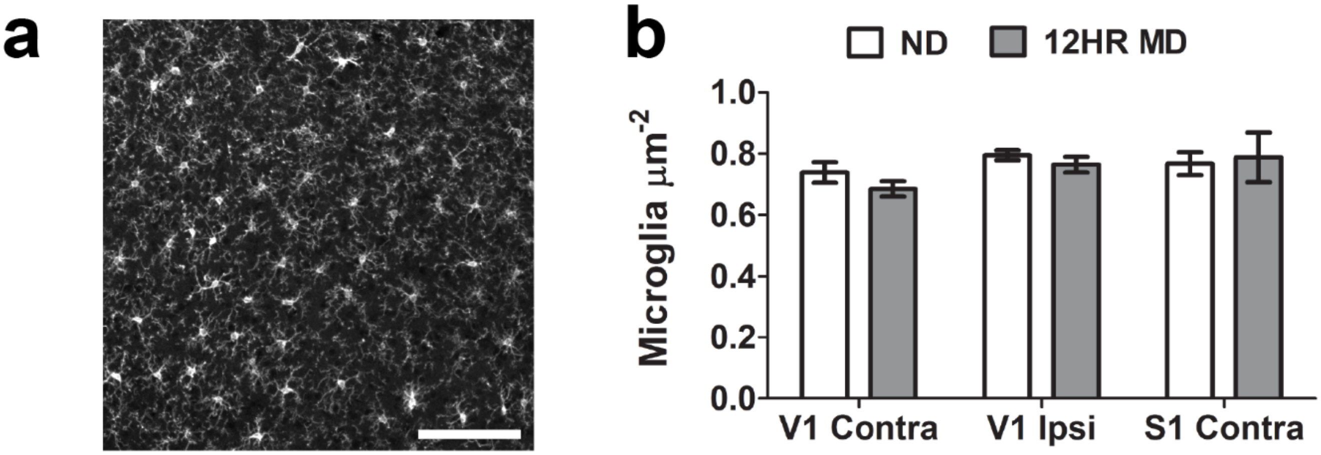

Supplementary Figure 2. Microglial density is unchanged by monocular deprivation

a. Image of Iba-1 immunoreactive microglia in the contralateral binocular visual cortex after 12hr MD. Scale bar = 100 µm. b. Microglial density was comparable between contralateral binocular visual cortex, ipsilateral binocular visual cortex and primary somatosensory cortex of the same animals. Densities were unchanged by 12 hours of MD in all three regions (n=4,4,4,4,5,4, One-way ANOVA, $F(1,19) = 0.3799$, $p=0.5450$). Graphs show mean +/- SEM.

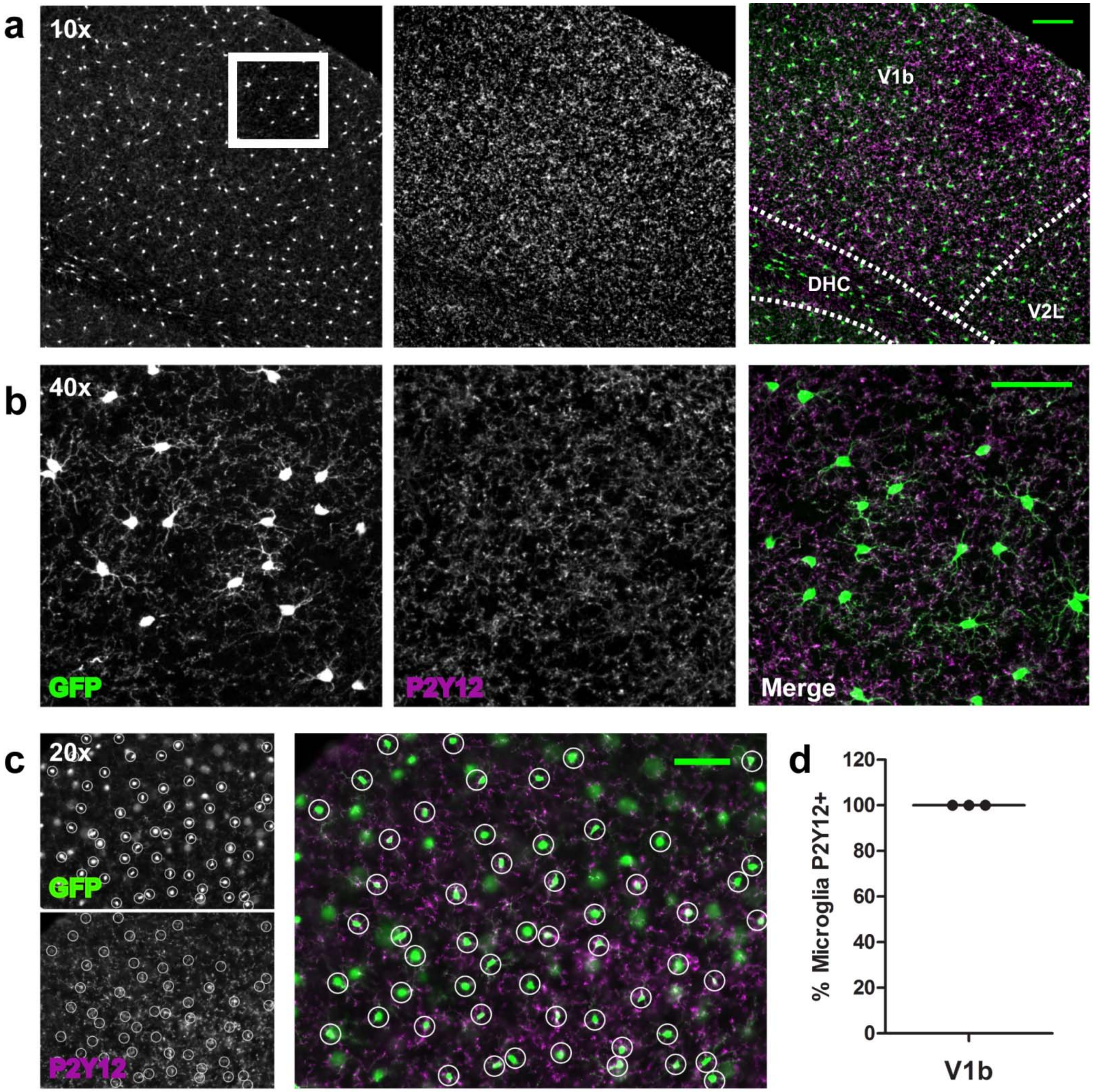

Supplementary Figure 3. P2Y12 is highly expressed in cortical microglia

a-b. Confocal images of P2Y12 immunoreactivity in V1b of *$CXC3CR1^{GFP/+}$* mice showing high expression on microglial processes. Images were taken using a 10x (a) and a 40x (b) objective. Square selection represents location of 40x image. Left panels show GFP fluorescence representing microglia, middle panels show P2Y12 immunoreactivity, and right panels represent the merge (GFP = green; P2Y12 = purple). c. High levels of co-localization between GFP and P2Y12 immunoreactivity in $CXC3CR1^{GFP/+}$ mice were observed in epifluorescence images.Images with circles denote in focus microglia counted for analysis. d. Graph showing quantification in three animals, where every microglia contained P2Y12 immunoreactivity. V1b: primary visual cortex binocular area. DHC: dorsal hippocampal commissure; V2L: secondary visual cortex lateral area. Scale bars = 100 µm (a); 50 µm (b); 50 µm (c).

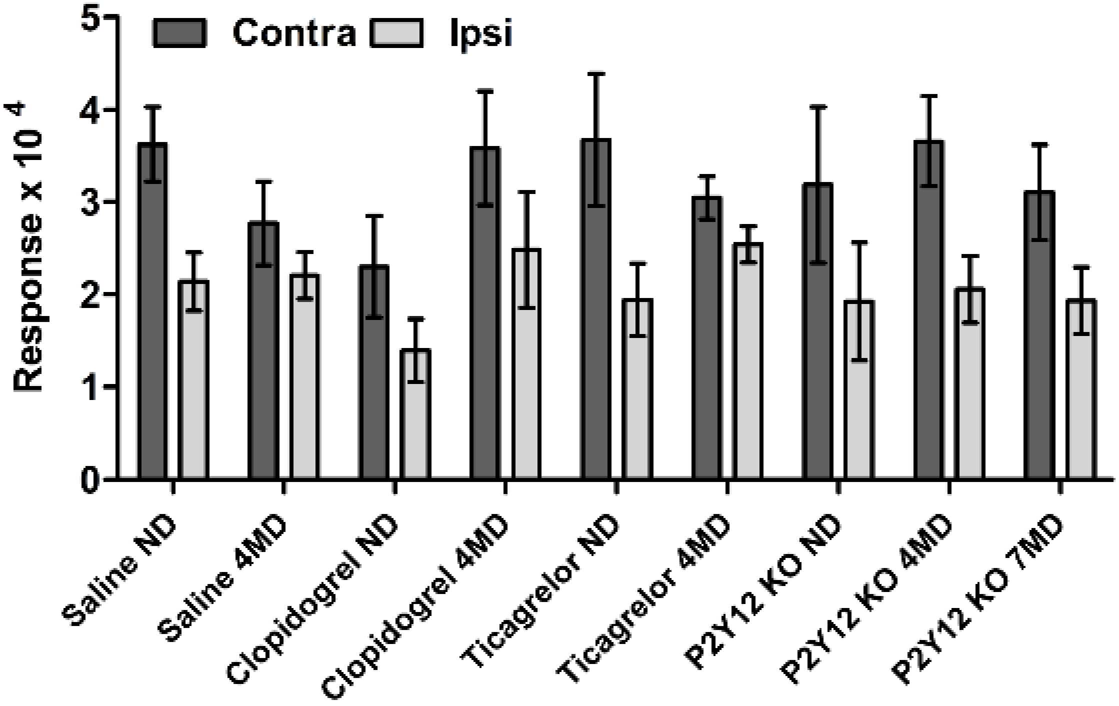

Supplementary Figure 4. Changes in single eye responses after MD

The response from the ipsilateral and contralateral eye is plotted for the different conditions corresponding to the ocular dominance index (ODI) shifts presented in Fig. 5f. Notice that after 4D MD there is a reduced response to stimulation of the contralateral eye in control saline-treated mice but no change in the ipsilateral response. This reduction does not occur in *P2Y12 KO* mice. Graphs show mean +/- SEM.

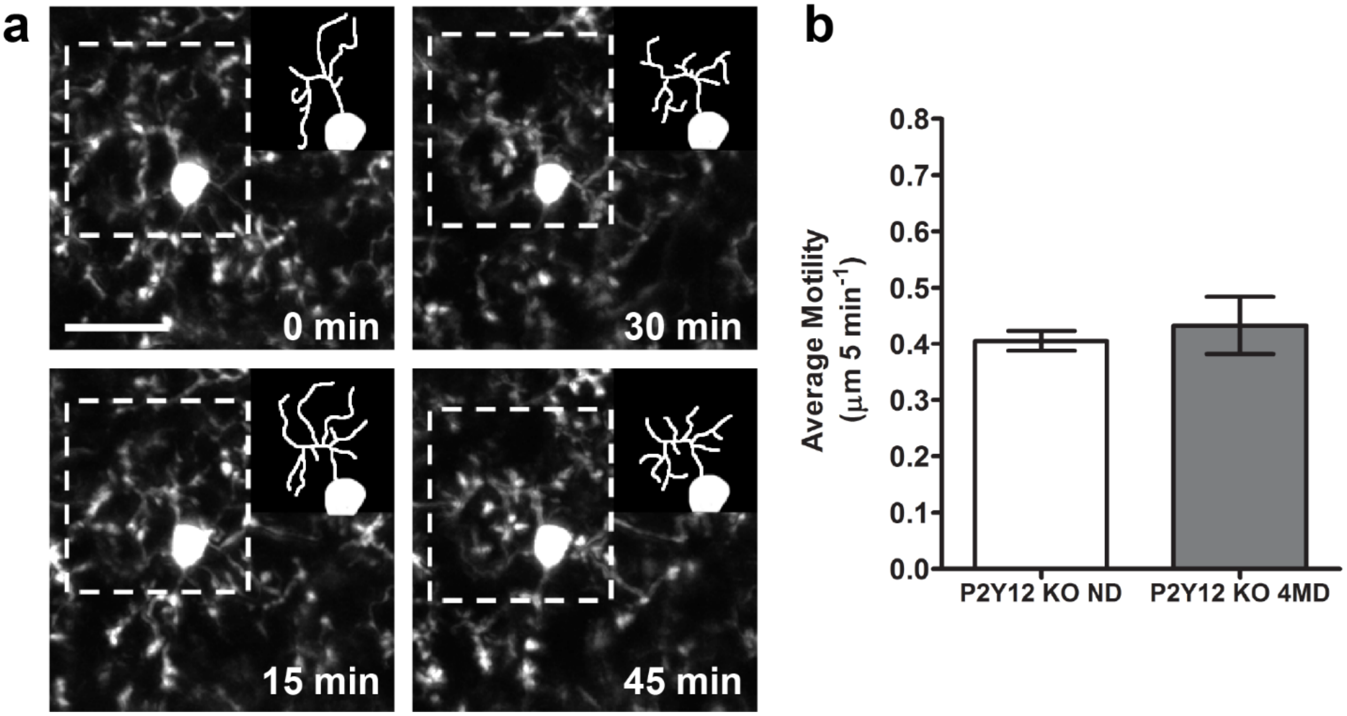

Supplementary Figure 5. Confirmation of a lack of change in microglial motility in P2Y12^{KO} mice after 4 days of monocular deprivation

a. Images showing microglia imaged *in vivo* in *P2Y12^{KO}* mice. Traced processes in the boxed area are shown in insets in the upper right-hand corner of each panel. b. Quantification of traced processes shows no change in motility after deprivation in *P2Y12^{KO}* mice (Student's t-test, $t(7) = 0.55$ $p=0.60$). Scale bar = 20 µm. Graphs show mean +/- SEM.

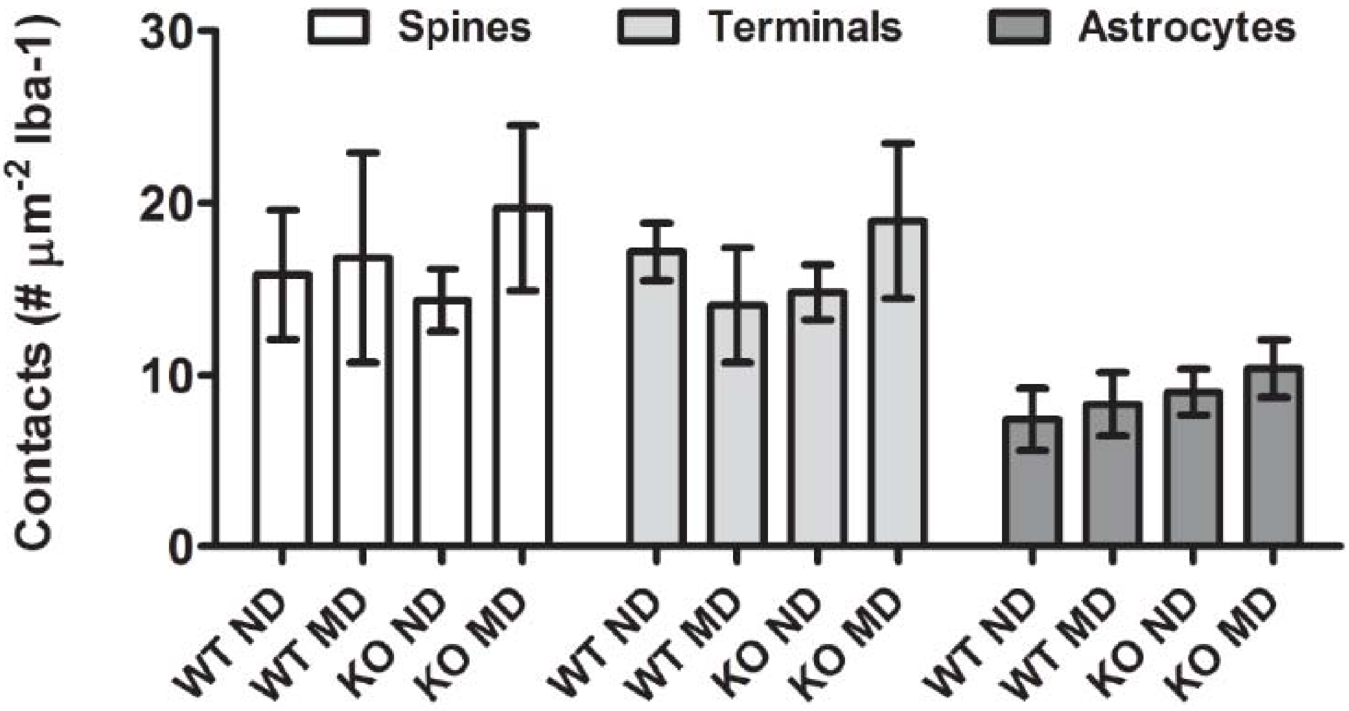

Supplementary Figure 6. Interactions between microglia and synaptic and perisynaptic elements after MD

There was no effect of deprivation on microglial contacts with dendritic spines, axon terminals or astrocytic processes in either *P2Y12 WT* or *KO* mice (n=5, p=0.95, F(2,36) = 0.049; two-way ANOVA). Graphs show mean +/- SEM.

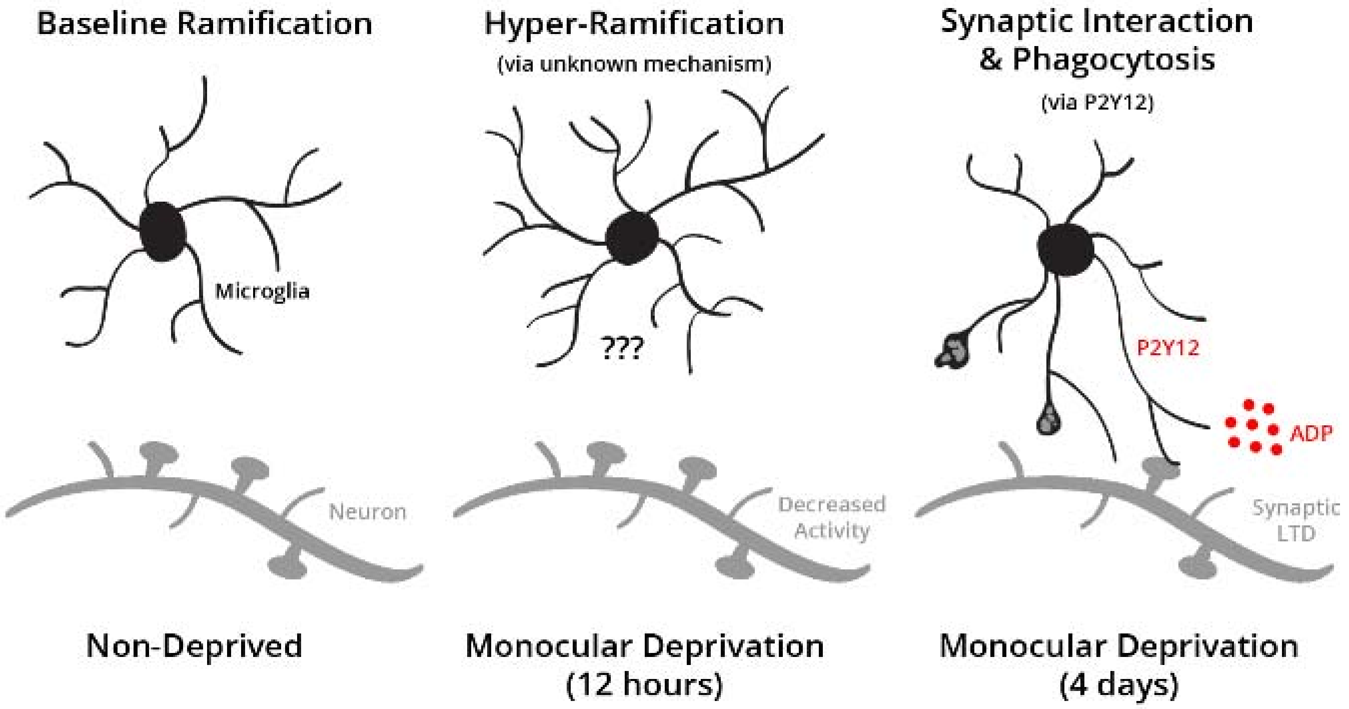

Supplementary Figure 7. Model of microglial actions during ODP

In non-deprived visual cortex, microglia are highly ramified and processes are motile. Within 12 hours after monocular deprivation, decreased activity in neurons leads to a rapid microglial hyper-ramification through an unknown mechanism. On a slower scale, ADP release from neurons or astrocytes activates P2Y12 on microglia, triggering microglial process targeting to depressed synapses, decreased process motility and phagocytosis of postsynaptic material.

Supplementary Table 1: Top: Holm-Sidak post-hoc comparisons of microglial morphology (Sholl analysis; Fig. 1b (top) and Fig. 1d (bottom)) after MD. Post hoc comparisons are made relative to the ND results from the same brain area. Notice that following 12 HR, 1D, 2D and 4D MD, significant hyper-ramification is observed between 20 and 32 μm from the soma (**p<0.05, **p<0.01, ***p<0.001, ****p<0.0001*).

MD Duration	Distance from Soma (μm)								
	18	20	22	24	26	28	30	32	34
12 HR MD	-	*	***	****	****	**	**	-	-
1D MD	-	**	***	****	****	****	****	*	-
2D MD	-	*	***	****	***	**	***	-	-
4D MD	-	-	-	-	*	-	*	-	-
7D MD	-	-	-	-	-	-	-	-	-

ND vs. 12HR MD	Distance from Soma (μm)												
	10	12	14	16	18	20	22	24	26	28	30	32	34
V1 Contra	-	***	****	****	****	****	****	****	****	****	****	**	-
V1 Ipsi	-	-	-	-	-	-	-	-	-	-	-	-	-
S1 Contra	-	-	-	-	-	-	-	-	-	-	-	-	-

Supplementary Table 2: Holm-Sidak post-hoc comparisons of microglial morphology (Sholl analysis; Fig. 7) in *P2Y12 WT* (top) and *P2Y12 KO* (middle) during MD. Notice significant hyper-ramification at 12 HR MD in both *P2Y12 WT* and *KO* mice. Holm-Sidak post-hoc comparisons of WT vs. KO mice (bottom) show that microglial ramification is significantly reduced before and during MD in KO mice (**p<0.05, **p<0.01, ***p<0.001, ****p<0.0001*).

P2Y12 WT

	Distance from Soma (μm)													
MD Duration	12	14	16	18	20	22	24	26	28	30	32	34	36	38
12 HR MD	-	*	****	****	****	****	****	****	****	****	****	****	**	-
2D MD	-	-	-	-	-	-	-	-	-	-	-	-	-	-
4D MD	-	-	-	-	-	-	-	-	-	-	-	-	-	-
7D MD	-	**	*	*	***	***	**	-	-	-	-	-	-	-

P2Y12 KO

	Distance from Soma (μm)														
MD Duration	6	8	10	12	14	16	18	20	22	24	26	28	30	32	34
12 HR MD	-	*	**	****	****	****	****	****	****	****	****	****	****	*	-
2D MD	-	-	-	-	-	-	*	-	-	-	-	-	-	-	-
4D MD	-	-	-	-	-	-	-	-	-	-	-	-	-	-	-
7D MD	-	-	-	-	-	-	-	-	-	-	*	-	-	-	-

WT vs. KO

	Distance from Soma (μm)										
MD Duration	16	18	20	22	24	26	28	30	32	34	36
ND	-	*	**	**	**	-	-	-	-	-	-
12 HR MD	-	-	-	**	***	****	***	****	***	**	-
2D MD	-	-	-	-	-	-	-	-	-	-	-
4D MD	-	*	****	****	****	****	****	*	-	-	-
7D MD	-	*	*	**	***	***	**	-	-	-	-

Supplementary Table 3: Sex distributions of mice used in experiments listed by figure number. Please note that for experiments in Fig. 6 not all information is available.

Condition	# ♂	# ♀	Condition	# ♂	# ♀
**Fig. 1 a,b,e,f**			**Fig. 5 a-b**		
ND	2	2	saline	2	3
12hr MD	2	1	clopidogrel	4	1
1D MD	1	2	**Fig. 5 c-d**		
2D MD	2	2	WT	2	2
4D MD	2	2	P2Y12 KO	3	2
7D MD	1	2	**Fig. 6; S. Fig. 5**		
**Fig. 1 c,d; SFig. 2**			WT	6	2
contra V1b ND	2	2	P2Y12 KO	8	0
contra V1b 12hr MD	3	3	**Fig. 7 a-c**		
ipsi V1b ND	2	2	WT ND	5	1
ipsi V1b 12hr MD	2	3	WT 12hr MD	4	2
S1 ND	3	1	WT 2d MD	4	2
S1 12hr MD	2	2	WT 4d MD	3	3
**Fig. 2b**			WT 7d MD	2	4
2D ND	5	3	P2Y12 KO ND	2	4
2D MD	3	3	P2Y12 KO 12hr MD	1	5
4D ND	2	2	P2Y12 KO 2d MD	3	3
4D MD	2	4	P2Y12 KO 4d MD	6	0
**Fig. 2d**			P2Y12 KO 7d MD	0	6
ND	2	3	**Fig. 7 d-e**		
MD	3	2	P2Y12 KO ND	2	3
**Fig. 3 e-g**			P2Y12 KO 4d MD	4	1
ND	2	3	**Fig. 8 a-c; S. Fig. 6**		
4d MD	3	2	WT ND	4	2
**Fig. 4c**			WT 4d MD	4	2
saline	2	1	P2Y12 KO ND	2	4
clopidogrel	1	2	P2Y12 KO 4d MD	3	3
tricagelor	2	3	**Fig. 8. d-f**		
**Fig. 4f; S. Fig. 4**			P2Y12 KO ND	2	2
saline ND	0	5	P2Y12 KO 4d MD	2	2
saline 4d MD	6	1	**S. Fig. 1**		
clopidogrel ND	4	2	ND	3	2
clopidogrel 4dMD	3	2	12hr MD	3	2
tricagelor ND	2	3	1D MD	2	2
tricagelor 4dMD	2	4	2D MD	2	2
P2Y12 KO ND	5	1	4D MD	2	3
P2Y12 KO 4dMD	5	0	7D MD	3	1
P2Y12 KO 7dMD	4	0			
